# Loss of Arabidopsis ACR11 results in altered C/N balance and high sensitivity to nitrogen toxicity

**DOI:** 10.3389/fpls.2025.1679652

**Published:** 2025-12-01

**Authors:** Jacob R. Kunkel, Hiroshi A. Maeda, Jorge El-Azaz

**Affiliations:** Department of Botany, University of Wisconsin - Madison, Madison, WI, United States

**Keywords:** amino acid metabolism, nitrogen metabolism, nitrogen assimilation, ACT domains repeats, carbon/nitrogen balance

## Abstract

**Introduction:**

Plants invest large amounts of resources to produce the twenty proteinogenic amino acids that are essential for growth. However, we still lack a comprehensive understanding of the regulation of amino acid metabolism during the plant life cycle. Plants have a highly conserved ACT domain repeats (ACR) family proteins, which are structurally similar to the bacterial sensor protein GlnD that regulates a key enzyme for nitrogen assimilation and amino acid biosynthesis, glutamine synthetase (GS).

**Methods:**

We investigated the role of the plastidial ACR proteins *acr11* and *acr12* in the regulation on amino acid metabolism by quantifying the levels of amino acids and other metabolites in Arabidopsis *acr11* and *acr12* knockout mutants grown under varying light and nitrogen fertilization conditions.

**Results:**

Unlike *acr12* plants, which showed only minor growth alterations, *acr11* mutants exhibited markedly delayed growth and carbon/nitrogen imbalance. At the metabolic level, *acr11* plants showed overaccumulation of free amino acids and other nitrogen-containing metabolites, particularly when grown under high nitrogen conditions. Further, *acr11* plants exhibited a marked decrease in the levels of keto acid intermediates from central carbon metabolism that are precursors to amino acid biosynthesis. Quantification of total GS activity, a potential regulatory target for *acr11* according to previous studies, shows similar levels of GS activity between *acr11* and Col-0 controls under the growth conditions tested here.

**Conclusions:**

Our findings suggest that *acr11* is a negative regulator of plant nitrogen metabolism that operates through a mechanism different from bacterial GlnD, possibly regulating other molecular targets besides plastidial GS.

## Introduction

Plants, unlike animals, synthesize *de novo* all twenty proteinogenic amino acids starting from CO_2_ and inorganic nitrogen, which is assimilated into glutamine and glutamate via the glutamine synthetase/glutamine α-ketoglutarate amidotransferase (GS/GOGAT) cycle. The reduced nitrogen harbored by these two amino acids can then be transferred across the plant metabolic network to synthesize the proteinogenic amino acids—whose biosynthetic pathways are predominantly plastidial—among other essential nitrogen-containing metabolites. Nitrogen assimilation and amino acid biosynthesis are energy-intensive processes, and as such are highly regulated (Less and Galili, 2008; Galili et al., 2016; Pratelli and Pilot, 2014; Arnold et al., 2015). A cornerstone element of control over amino acid metabolism in both plants (Curien et al., 2008; Jander and Joshi, 2009; Binder, 2010; Maeda and Dudareva, 2012; Galili et al., 2016) and microorganisms (Sander et al., 2019; Naz et al., 2023) is feedback regulation of key enzymatic activities in response to metabolites. This regulation is often allosteric, mediated by specific regulatory domains that bind amino acid(s), typically the amino acid(s) produced in that pathway, which act as allosteric effector(s) that induce conformational changes in the enzyme and change its activity level. Feedback regulation provides an efficient mechanism to harmonize different pathways based on sensing the levels of certain metabolites, and adjusting key enzymatic activities accordingly.

One of the most widespread allosteric regulatory domains involved in the feedback regulation of amino acid biosynthetic pathways is the ACT domain, found in both plants and microorganisms. Characterized by a βαββαβ topology, the ACT domain was first reported in the *Escherichia coli* enzymes Aspartate Kinase, Chorismate mutase, and TyrA (from which its name derives) (Grant, 2006). Although widespread across enzymes involved in amino acid biosynthesis (Liberles et al., 2005; Grant, 2006), ACT-like domains can also be found in proteins having other functionalities. These include plant-unique transcription factors in the basic helix-loop-helix (bHLH) family (Lee et al., 2023a) or the family of serine/threonine/tyrosine (STY) kinases. These latter regulate access to plastids of some nucleus-encoded proteins depending on isoleucine and *S*-adenosylmethionine levels (Eisa et al., 2019) and control ammonium (NH_4_^+^) uptake via phosphorylation of key transporters (Beier et al., 2018; Straub et al., 2017). Thus, ACT domains are fundamental to control amino acid metabolism, either by directly mediating feedback regulation of enzymes, or by acting as molecular sensors that ignite more sophisticated cellular responses.

One of multiple examples of ACT domain-containing molecular sensor is the bacterial protein GlnD, a bifunctional uridylyltransferase/uridylyl-removing enzyme that is allosterically regulated by the levels of glutamine and α-ketoglutarate via two C-terminal ACT domains (Adler et al., 1975; Brown et al., 1971; Garcia and Rhee, 1983; Stadtman, 2004, Leigh and Dodsworth, 2007; Bolay et al., 2018). Under high glutamine versus α-ketoglutarate levels (which indicates high nitrogen status in the cell), GlnD operates as a uridylyl-removing enzyme and de-uridylates the regulatory protein P_II_, which in turn adenylates GS, reducing GS activity and slowing down glutamine biosynthesis. Conversely, under low nitrogen status, α-ketoglutarate becomes more abundant than glutamine and binds to GlnD, promoting GlnD uridylyltransferase activity over P_II_. This uridylated P_II_ de-adenylates GS, making this enzyme more active and enhancing glutamine biosynthesis.

Interestingly, plant genomes harbor a family of ACT domain containing proteins having structural resemblance to bacterial GlnD regulatory protein. This family, known as ACT domain repeats (ACR), is formed by twelve members in Arabidopsis (ACR1-12; Sung et al., 2011). Among them, ACR11 and ACR12 are targeted to plastids, where the bulk of nitrogen assimilation and amino acid biosynthesis takes place. In agreement with the potential regulatory role of plant’s ACR proteins over amino acid metabolism, Arabidopsis knock-out mutants for *ACR11* show altered levels of free amino acids and delayed growth (Takabayashi et al., 2016; Osanai et al., 2017). Moreover, these studies have proposed that ACR11 interacts with the plastidial GS of plants (encoded by *GLN2*) (Osanai et al., 2017) and the plastidial ferredoxin-dependent GOGAT (Fd-GOGAT) (Takabayashi et al., 2016). However, the exact function and mechanism of ACR11 regulation, as well as the other plant ACR proteins, remains unclear.

In this study, we have investigated the role of ACR11 and ACR12 in amino acid biosynthesis by conducting targeted and untargeted metabolic analysis of Arabidopsis knock-out mutants from both genes, grown under various light intensities, environmental CO_2_ concentration and nitrogen fertilization regimes. Whereas little changes were observed in *acr12* mutants besides moderate growth reduction, *acr11* plants showed altered carbon/nitrogen balance and overaccumulated multiple amino acids and other nitrogen-containing metabolites, whilst having decreased levels of α-ketoglutarate and other tricarboxylic acid (TCA) intermediates. These metabolic alterations were exacerbated by high nitrogen fertilization, which caused severe toxicity in *acr11* plants. Taken together, these findings revealed that knock-out mutation of *ACR11* causes overproduction of nitrogen-containing molecules at the cost of depleting key intermediates of central carbon metabolism, supporting that *ACR11* is an important negative regulator of nitrogen metabolism in plants.

## Results

### Plastid localized ACR11 and ACR12 are conserved across Viridiplantae

**Figure 1:**
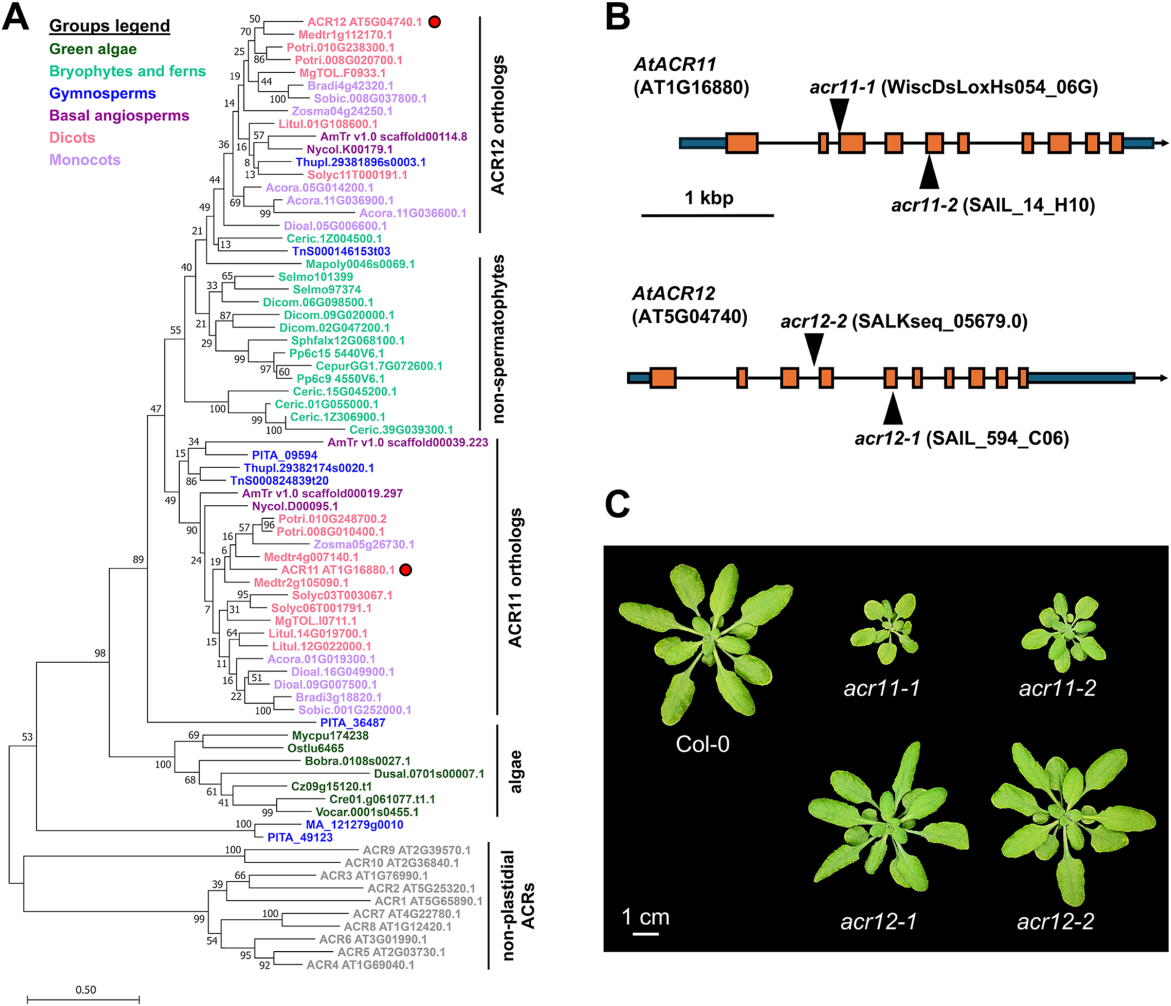
Plastidial ACR genes are widely conserved throughout the green lineage. **(A)** Phylogeny of plastidial ACR proteins in plants and green algae. Arabidopsis ACR11 and ACR12 are marked with a red dot. Non-plastidial ACR proteins from Arabidopsis (ACR1 to 10, grey color) were set as outgroup. **(B)** Arabidopsis ACR11 (AT1G16880) and ACR12 (AT5G04740) gene structures, with T-DNA insertions. Orange boxes represent exons, black lines represent introns, and dark blue boxes represent UTR regions. T-DNA mutant lines were selected and named according to previous studies (Osanai et al., 2017). **(C)** Image of one-moth-old Arabidopsis Col-0, grown side-by-side together with *acr11* and *acr12* knock-out mutants under standard growth conditions: ~100 µmol·m^-2^·s^-1^ light intensity, 12h day/night cycle at 22 °C/18 °C, 70% humidity, and watered regularly with a 1:10 dilution of Hoagland’s solution in distilled water (see further details in methods).

To investigate the evolutionary origins of plastid localized ACR proteins, we reconstructed a phylogeny of AtACR11 and AtACR12 orthologs across land plants and green algae (**Figure 1A**). This phylogeny supported that angiosperm and gymnosperm (i.e., spermatophytes) genomes generally harbor at least one ortholog for each ACR11 and ACR12 (**Figure 1A**), >80% of them having a predicted plastid transit peptide (**Supplementary S1**). Bryophytes and fern species have between one and five ACR11/12 related sequences, located in an uncertain position in the phylogeny between the ACR11 and ACR12 clades of embryophytes (**Figure 1A**), with variable subcellular localization predictions between plastid and cytosol (**Supplementary S1**). Conversely, only a single copy ortholog gene was found in algae species (**Figure 1A**), having predicted plastidial localization (**Supplementary S1**). These observations indicate that the proteins encoded by *ACR11* and *ACR12* are plastid-localized across embryophytes, suggesting that these genes come from a single copy algae gene that underwent duplication and sequence divergence during the evolution of land plants.

### Arabidopsis *acr11* mutants, but not *acr12*, show reduced growth that is influenced by light

Next, we isolated two full knock-out T-DNA Arabidopsis insertion mutant lines for *ACR11* and *ACR12*: *acr11-1* (WiscDsLoxHs054_06G), *acr11-2* (SAIL_14_H10), *acr12-1* (SAIL_594_C06), and *acr12-2* (SALKseq_05679.0) (**Figures 1B, C**). These lines were named following the same nomenclature as in Takabayashi et al., 2016, which reported increased amino acid levels in *acr11–1* and *acr11-2*. LC-MS analysis of Col-0, *acr11–1* and *acr11–2* mutant lines to measure the free levels of 19 proteinogenic amino acids (all but cysteine, which could not be detected in the LC-MS method used) confirmed that both *acr11* knock-out mutants overaccumulate multiple amino acids compared to Col-0 wild-type (**Supplementary S2**). As both independent *acr11–1* and *acr11–2* knock-out lines shared this phenotype, we selected line *acr11–1* to conduct further experiments.

**Figure 2:**
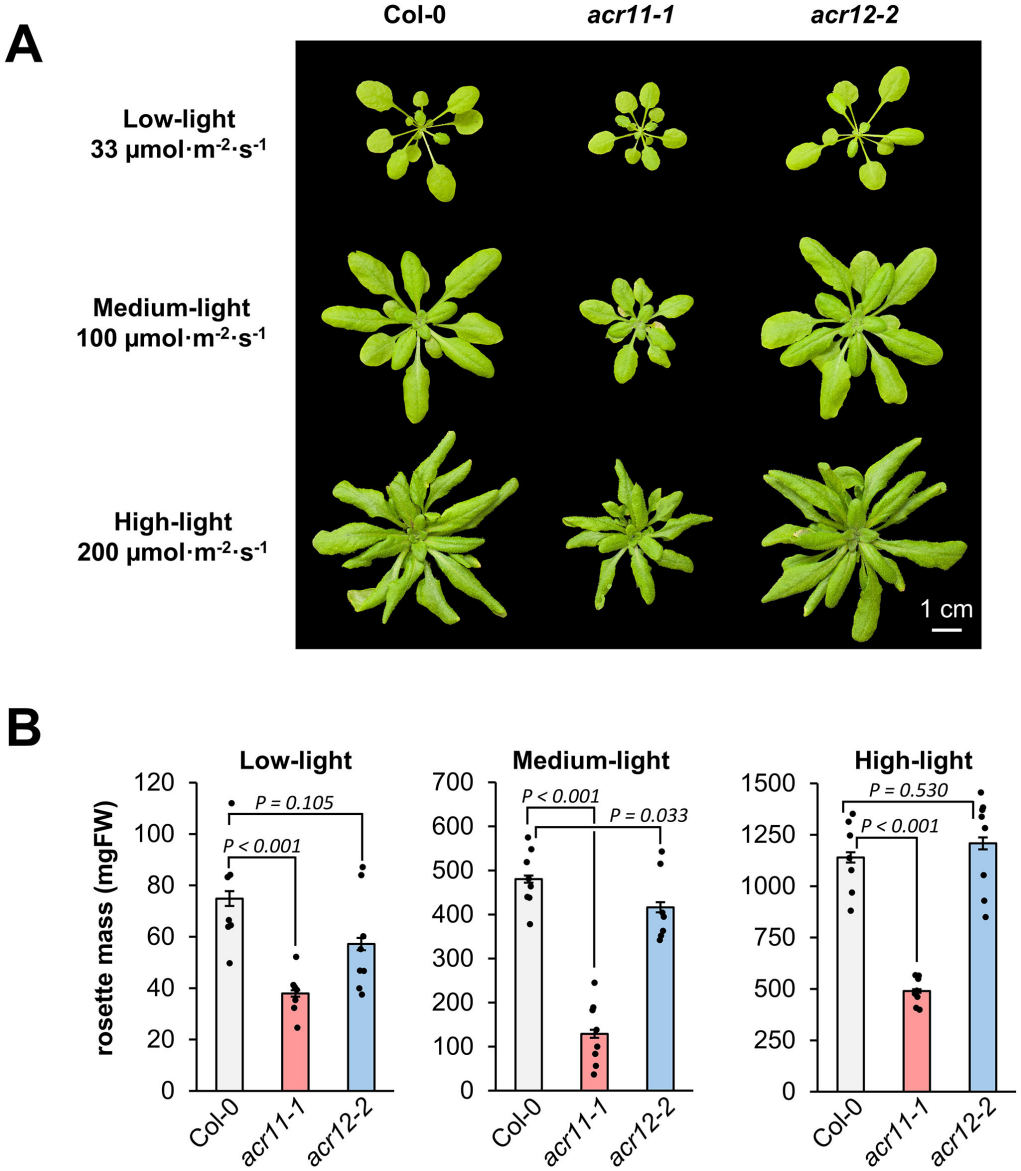
Growth of *acr11* mutants is reduced more strongly at around 100 µmol·m^-2^·s^-1^ light intensity than at lower or higher light intensities. **(A)** One-month-old Col-0, *acr11–1* and *acr12–2* plants grown side-by-side on soil at three alternative light intensities. **(B)** Average total rosette mass, in milligrams of fresh weight (mg FW), of the plant groups shown in **(A)** Bar graphs represent the average of *n* = 7–8 individual plants; each individual plant is represented by a black dot. Error bars correspond to standard error. *P*-values are indicated over each bar graph according to Student’s t-test (two tailed test, equal variance).

Knock-out mutation of *ACR11* has been previously described to negatively impact Arabidopsis growth, and that light intensity affects this growth phenotype (Osanai et al., 2017). To investigate whether different light conditions may affect *acr11* and *acr12* growth, we grew the mutant lines *acr11–1* and *acr12–2* together with Col-0 plants for one month under low-, medium- and high-light intensities, corresponding to around 33, 100 and 200 µmol·m^-2^·s^-1^, respectively. As reported before (Osanai et al., 2017), the stunted growth of *acr11–1* mutants was significantly affected by light intensity (*P*-value < 0.001; one-way ANOVA). Under medium-light intensity, *acr11–1* reached only ~25% of the rosette mass of Col-0 plants (**Figure 2A**), often showing leaves with necrotic lesions during the first two weeks after germination. Under low- and high-light, the relative growth of *acr11–1* plants compared to Col-0 was less reduced than in medium-light, reaching ~40-50% of the total rosette mass of Col-0 (**Figure 2B**). Conversely, *acr12–2* plants had no obvious growth defects compared to Col-0 under any light conditions tested (**Figures 2A, B**), besides a trend towards reduced growth in low- and medium-light (**Figure 2B**). Hence, the reduced growth of *acr11* is more apparent around the light intensity frequently used for Arabidopsis growth (i.e., ~100 µmol·m^-2^·s^-1^), with both lower and higher light intensities partially attenuating this phenotype.

### Overaccumulation of free amino acids in *acr11* positively correlates with light intensity

**Figure 3:**
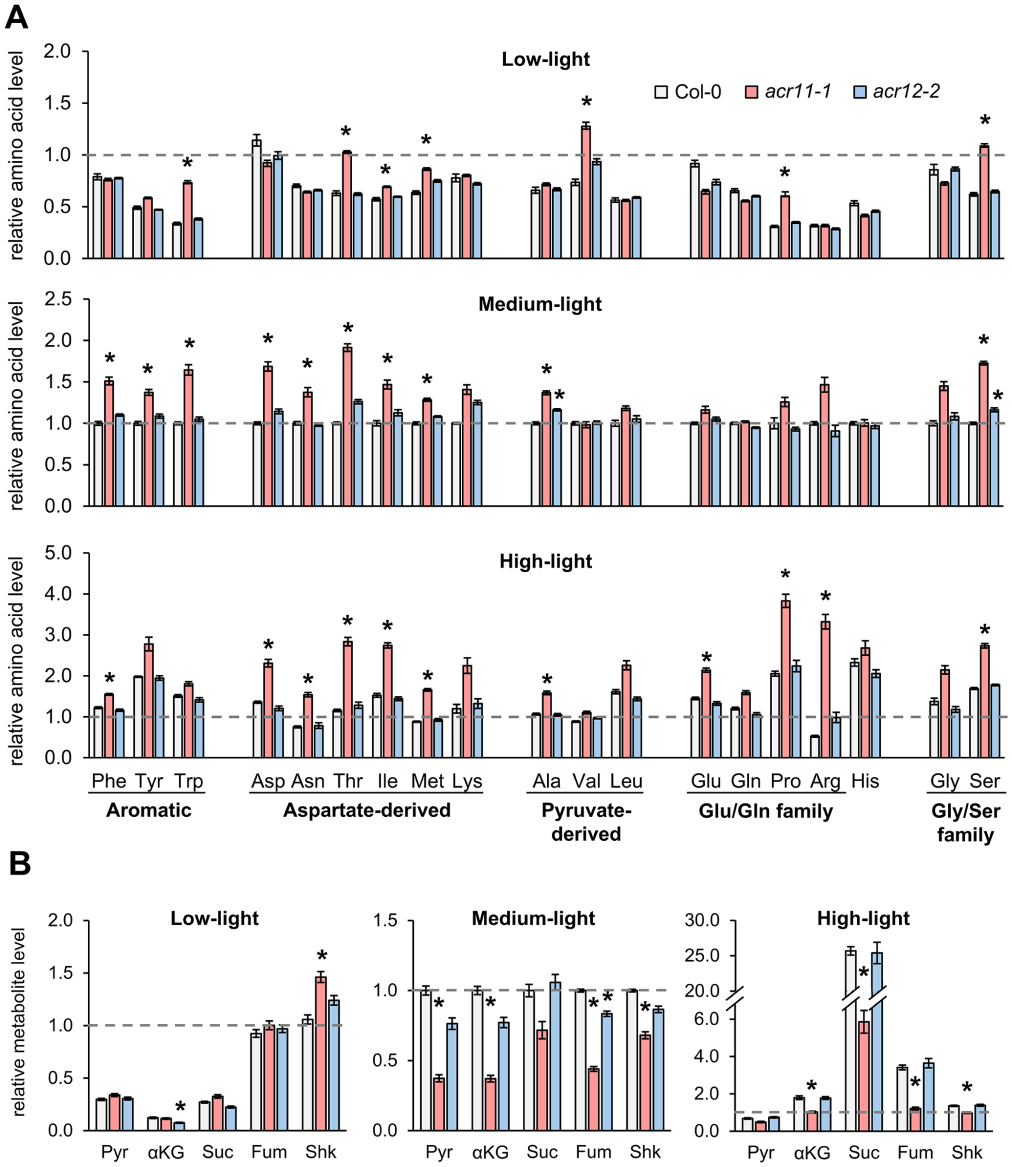
Knock-out mutation of *ACR11*, but not *ACR12*, causes overaccumulation of multiple amino acids and depletion of TCA cycle intermediates. **(A)** Levels of 19 free proteinogenic amino acids (all proteinogenic amino acids but cysteine) in whole rosettes of one-month-old Col-0, *acr11–1* and *acr12–2* plants grown under alternative light intensities in a Percival growth chamber (see methods). Amino acids named following the standard three-letter code. **(B)** Levels of pyruvate (Pyr), α-ketoglutarate (αKG), succinate (Suc), fumarate (Fum) and shikimate (Shk) in the same plant samples. Metabolite levels were determined by LC-MS using authentic standards, and expressed in relative units by assigning the value of “one” (indicated by horizontal dashed line on each graph) to the average level of that metabolite in Col-0 plants grown under standard-light conditions. Bar graphs represent the average of *n* = 7–8 individual plants. Error bars correspond to standard error. Asterisks indicate significant differences compared to Col-0 plants grown under the same light intensity, according to *post hoc* ANOVA Tukey’s test (*α* = 0.05).

To explore whether light-dependent changes in *acr11* growth may be linked to amino acid metabolism, we profiled free amino acid levels in the rosette leaves of one-month-old Col-0, *acr11–1* and *acr12–2* plants grown under low-, medium- and high-light conditions. Whereas we detected little changes in *acr12–2* plants compared to Col-0, and these changes were not statistically significant altogether (*P*-value = 0.319; two-ways ANOVA) (**Figure 3A**), *acr11–1* plants overaccumulated multiple amino acids in a light-influenced manner (*P*-value < 0.001; two-ways ANOVA). According to *post hoc* ANOVA Tukey’s test (*α* = 0.05), seven amino acids (tryptophan, threonine, isoleucine, methionine, valine, proline and serine) were significantly overaccumulated in *acr11–1* versus Col-0 under low-light, compared to 10 overaccumulated amino acids under medium-light, and 11 under high-light (**Figure 3A**). Although multiple amino acids coming from independent biosynthetic pathways were upregulated in *acr11–1* in at least one of the light treatments tested (**Figure 3A**), the aspartate-derived amino acids (e.g., threonine, isoleucine) were particularly affected across multiple conditions (**Figure 3A**). Higher light intensities also caused higher fold changes in individual amino acid levels, with arginine—the most nitrogen-rich amino acid—becoming 6-times more abundant in *acr11–1* compared to Col-0 under high-light (**Figure 3A**). In contrast with prior studies reporting lower glutamine levels in *acr11* (Osanai et al., 2017), glutamine levels were unchanged between *acr11–1* and Col-0, except for a 1.3-times increase under high-light conditions, although on the limit of statistical significance (*P*-value = 0.06, *post hoc* ANOVA Tukey’s test) (**Figure 3A**). Despite clear overaccumulation of multiple amino acids in *acr11*-*1*, we observed a tendency towards reduced glutamate levels under low-light (**Figure 3A**). In brief, these findings show that *acr11* plants overaccumulate multiple amino acids, especially when grown under higher light intensity.

Given that many proteinogenic amino acids are upregulated in *acr11*, we hypothesized that *acr11* plants may also exhibit altered levels of key amino acid biosynthetic precursors, such as pyruvate (precursor to alanine and the branched chain amino acids valine and leucine; Binder, 2010), the intermediates of the tricarboxylic acid (TCA) cycle (i.e., α-ketoglutarate, succinate, fumarate), and shikimate (precursor to the three aromatic amino acids; Maeda and Dudareva, 2012). LC-MS quantification showed that these metabolites had altered levels in *acr11–1* in a light-influenced manner (*P*-value < 0.001; two-ways ANOVA). Under low-light, *acr11–1* plants exhibited no significant changes compared to Col-0 except for a ~40% increase in shikimate levels (**Figure 3B**). However, at medium-light intensity, these compounds experienced marked reductions in *acr11–1* compared to Col-0: around a 60% reduction for pyruvate, α-ketoglutarate, and fumarate, and around a 30% reduction for shikimate (**Figure 3B**). Under high-light, this reduction was even stronger for succinate and fumarate, which were down by around 75% and 65%, respectively, in *acr11–1* compared to Col-0 (**Figure 3B**). Despite this relative reduction in *acr11–1* versus Col-0 under high-light, it must be noted that these compounds were much more abundant in both *acr11–1* and Col-0 if compared to low- and medium-light (**Figure 3B**). Increased total levels of succinate, fumarate and other metabolites of central carbon metabolism under high-light may explain why this light treatment seemingly attenuates *acr11–1* reduced growth phenotype (**Figures 2A, B**) regardless of enhanced amino acid overaccumulation (**Figure 3A**). In contrast to *acr11-1*, *acr12–2* mutant showed limited changes compared to Col-0 with no significant effect from light intensity (*P*-value = 0.091; two-ways ANOVA) (**Figure 3B**). Thus, overaccumulation of free amino acids in *acr11* may be depleting the pools of various intermediates from central carbon metabolism that are precursors to amino acid biosynthesis.

### Untargeted metabolomics reveal overaccumulation of nitrogen rich compounds in *acr11* plants

**Figure 4:**
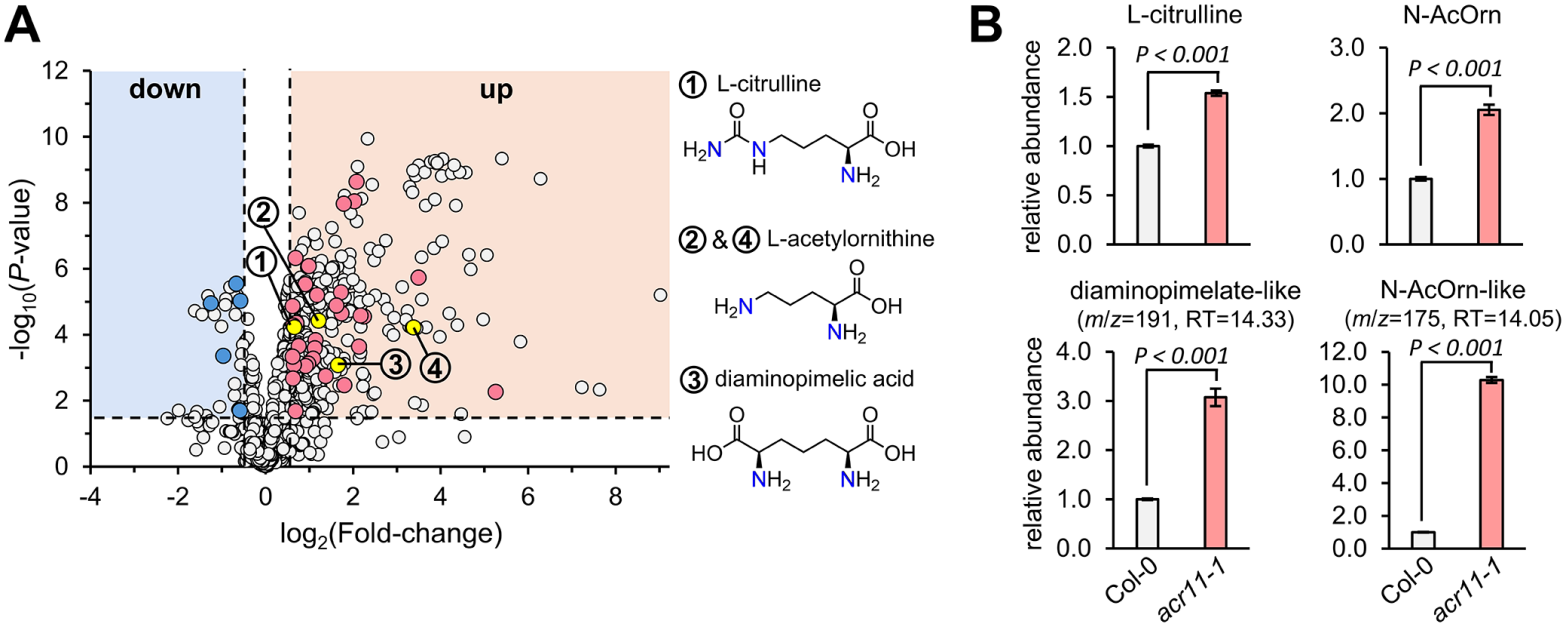
Untargeted metabolomics reveals overaccumulation of multiple nitrogen-containing metabolites in *acr11* plants. **(A)** Volcano plot comparing total mass features in the leaves of Col-0 and *acr11–1* plants grown on soil for one month under standard conditions (see methods). Each dot corresponds to the average level of a mass feature (*n* = 7), expressed as peak area divided by the mass of the sample in mg of fresh weight. Mass features only detected in one of the two genotypes were assigned an arbitrary value of 10 to determine fold-change between genotypes. Dots marked in blue or red correspond to non-redundant mass features having a KEGG access number, and thus being biologically relevant (see full list on **Table 1**). **(B)** Targeted metabolomics validation of the amino acid biosynthetic intermediates L-citrulline, N-acetylornithine (N-AcOrn) and diaminopimelate. The identity of the two upper panels, corresponding to L-citrulline and N-acetylornithine (N-AcOrn), was confirmed with authentic standards. The two bottom panels, corresponding to two mass features predicted as diaminopimelate and N-acetylornithine, were confirmed to not be these molecules and thus are labeled as “-like”. Bar graphs represent the average of *n* = 7 individual plants. Error bars correspond to standard error. *P*-values according to Student’s *t*-test (two tailed test, equal variance) are indicated on each bar graph.

To detect additional metabolic changes in *acr11* plants, we used MS/MS data to conduct an untargeted metabolomics comparative analysis of rosette leaves from one-month-old *acr11–1* and Col-0 plants grown under medium-light conditions. This analysis detected a total of 1,484 mass features, from which 407 and 52 features were significantly more and less abundant, respectively, in *acr11–1* compared to Col-0 (*log*_2_ fold-change 0.5; *P*-value < 0.05; Student’s *t*-test) (**Figure 4A**). These statistically significant mass features were then curated manually to include only non-redundant features (i.e., those not sharing the same correlation group or retention time) having a KEGG COMPOUND identifier (therefore, relevant to biological systems). This narrowed down the list to 31 and 5 candidate compounds that were more and less abundant, respectively, in *acr11–1* compared to Col-0 (**Figure 4A**; **Table 1**). Compounds enriched in *acr11–1* included six proteinogenic amino acids (already detected as elevated in *acr11* plants in previous experiments; **Figure 3A**, **Supplementary S2**) together with numerous predicted structures containing nitrogen, such as polyamines, acetylated amino acids, and various intermediates of amino acid pathways (e.g., L-histidinol, **Table 1**). Notably, 34 out of these 36 features were predicted to contain nitrogen (**Table 1**).

**Table 1:** Significantly changed non-redundant mass features and best molecular predictions in *acr11* plants.

Best prediction	*m*/*z* [M+H]+	RT (min)	Col-0 abundance	*acr11–1 abundance*	F-change (*acr11*/ Col-0)	P-value	Pred. score	Valid.
N-(1-carboxypropyl)glutamine	233.1129	15.56	4.3 ± 0.2	86 ± 10	19.7	0.013	0.15	
N-Acetyl-DL-leucine	174.1125	2.49	48 ± 0.9	489 ± 16	10.1	<0.001	0.28	
**N-Acetylornithine**	**175.1078**	**14.05**	**1326 ± 104**	**13641 ± 773**	**10.3**	**<0.000**	**0.26**	**False**
N-Acetylputrescine	131.1180	10.99	184 ± 8.7	913 ± 41	5.0	<0.001	0.53	
4-Coumaroylagmatine	277.1660	8.07	1180 ± 77	5369 ± 199	4.5	<0.001	0.99	
N6-acetyl-L-Lysine	189.1234	13.75	37 ± 1.5	137 ± 7.1	3.7	<0.001	0.55	
2-Benzanine, Isoquinoline	130.0652	6.72	4749 ± 194	19913 ± 292	4.2	<0.001	0.08	
4-Hydroxyquinoline	146.0602	12.96	254 ± 8.4	1075 ± 20	4.2	<0.001	0.17	
L-Histidinol	142.0976	13.36	170 ± 6.2	574 ± 42	3.4	0.004	0.76	
L-Homocysteic acid	184.0275	14.90	44 ± 0.7	151 ± 3.0	3.4	<0.001	0.53	
D-Hexose, Hexopyranose	145.0496	3.55	nd	127 ± 9.7	–	<0.001	0.32	
Feruloylagmatine	307.1764	7.92	72 ± 5.2	264 ± 9.2	3.6	<0.001	0.70	
**Diaminopimelic acid**	**191.1027**	**14.33**	**190 ± 3.3**	**586 ± 33**	**3.1**	**0.001**	**0.39**	**False**
N,N-dimethylarginine	203.1504	16.58	296 ± 7.9	893 ± 29	3.0	<0.001	0.33	
Sarcine, Hypoxanthine	137.0459	2.91	403 ± 14	1037 ± 57	2.6	0.002	0.08	
**N-Acetylornithine**	**175.1078**	**15.17**	**123 ± 3.0**	**254 ± 9.7**	**2.0**	**<0.000**	**0.20**	**True**
N(gamma)-Acetyldiaminobutyrate	161.0922	13.64	68 ± 1.4	160 ± 2.9	2.3	<0.001	0.19	
(-)-12-Hydroxyjasmonic acid	227.1278	2.64	63 ± 3.3	207 ± 9.0	3.3	<0.001	0.09	
Neoglucobrassicin	479.0791	4.94	350 ± 17	761 ± 24	2.2	<0.001	0.35	
L-Tryptophan; originally predicted as “3-indoleacrylate”	188.0707	10.12	226 ± 5.5	464 ± 18	2.0	<0.001	0.08	True
7-Methylguanine	166.0724	3.00	125 ± 1.8	243 ± 4.5	1.9	<0.001	0.09	
Allantoic acid	199.0437	14.25	65 ± 2.4	124 ± 4.5	1.9	0.001	0.64	
Indole-3-carboxylic acid	144.0445	2.78	278 ± 14	517 ± 14	1.9	0.001	0.31	
DL-Threonine	120.0657	13.22	5806 ± 103	10301 ± 169	1.8	<0.001	0.59	True
DL-Phenylalanine	166.0864	9.30	3744 ± 50	6403 ± 183	1.7	<0.001	0.99	True
DL-Aspartate	134.0449	15.99	2781 ± 24	4411 ± 48	1.6	<0.001	0.90	True
Scopoletin	193.0496	2.13	334 ± 26	549 ± 11	1.6	0.016	0.11	
**L-Citrulline**	**198.0850**	**15.05**	**1034 ± 14**	**1590 ± 28**	**1.5**	**<0.001**	**0.86**	**True**
L-Tyrosine; originally predicted as “p-Coumaric acid”	182.0813	11.53	270 ± 6.1	419 ± 12	1.5	0.001	0.07	True
8-methylthiooctyl glucosinolate	478.1237	4.16	1364 ± 26	2063 ± 24	1.5	<0.001	0.93	
DL-Serine	106.0502	14.03	2446 ± 56	3619 ± 92	1.5	0.001	0.53	True
4-Aminobenzoic acid	138.0550	2.38	739 ± 5.0	481 ± 7.0	0.65	<0.001	0.24	
Choline	104.1073	6.54	141011 ± 6145	94275 ± 3084	0.67	0.025	0.34	
Phosphocholine	184.0734	17.16	562 ± 9.6	359 ± 6.1	0.64	<0.001	0.87	
8-Hydroxyguanine	168.0517	6.20	217 ± 7.8	113 ± 3.5	0.52	0.001	0.12	
Melitose, Raffinose	527.1587	15.02	242 ± 7.1	97 ± 2.3	0.40	<0.001	0.10	

The list is limited to mass features having a KEGG identification number (thus being biologically relevant) and having a fold change (F-change) of >1.5-times in *acr11-1* compared to Col-0, for a *P*-value < 0.05 (*n* = 7 coming from independent plants) (Student’s t-test, two tailed distribution, equal variance). Compounds marked in bold were measured as part of main **Figure 4**. Pred. Score = score prediction from 0 to 1. Valid. = validation using authentic standards (False = not the predicted compound; True = confirmed to be the predicted compound; empty = not tested).

We then selected three candidate compounds for validation that were enriched in *acr11-1*: the lysine pathway intermediate diaminopimelic acid, and the arginine pathway intermediates L-citrulline and N-acetylornithine (this latter assigned to two independent mass features having the same *m*/*z* but different retention time; **Table 1**). Targeted LC-MS analysis using authentic pure standards confirmed L-citrulline and N-acetylornithine candidates eluting at 15.05 and 15.17 min, respectively (**Figure 4B****;****Supplementary S3**). The other candidate N-acetylornithine, eluting at 14.05 min, was thus not N-acetylornithine. The identity of candidate diaminopimelic acid (eluting at 14.33 min, **Table 1**) could not be confirmed, as the authentic diaminopimelic acid standard eluted at 17.05 min (**Supplementary S3**). These untargeted metabolite profiles support that *acr11* plants accumulate high levels of other nitrogen-bearing compounds besides proteinogenic amino acids.

### Disruption of ACR11 causes carbon/nitrogen imbalance

**Figure 5:**
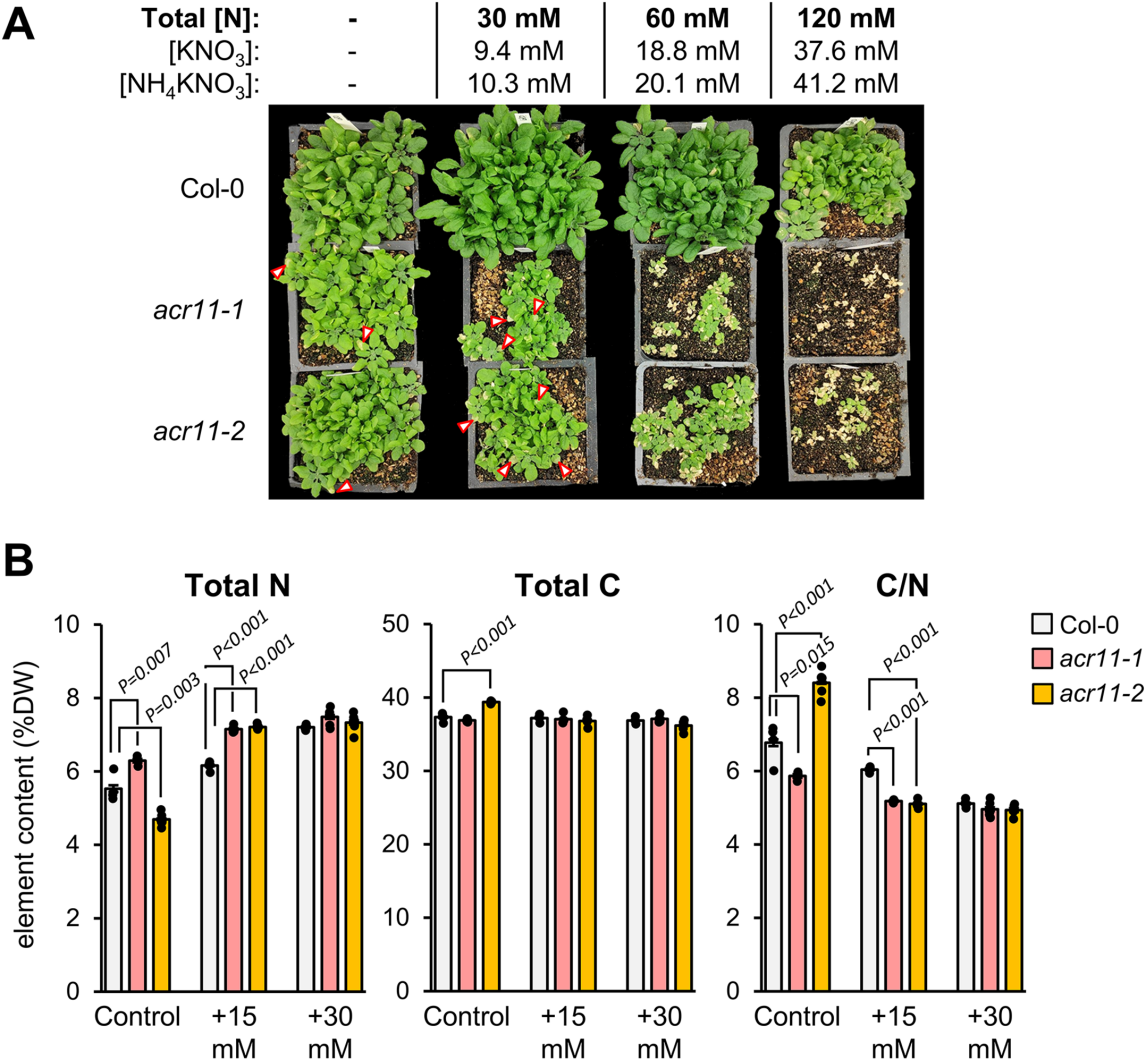
*acr11* plants experience severe toxicity under high nitrogen fertilization and show altered carbon/nitrogen ratios. **(A)** One-month-old Col-0, *acr11–1* and *acr11–2* plants after being regularly watered with solutions containing four alternative doses of total nitrogen, from 0 to 120 mM. Red arrows mark necrotic lesions on the leaves of *acr11–1* plants. **(B)** Determination of total elemental nitrogen, carbon, and carbon/nitrogen ratio, in the rosettes of Col-0, *acr11–1* and *acr11–2* plants watered with different nitrogen solutions. Bar graphs represent the average of *n* = 5 individual plants. Error bars correspond to standard error. Significant *P*-values according to Student’s *t*-test (*P* < 0.05, two tailed test, equal variance) are indicated on each bar graph; *P*-values above 0.05 (not significant) are not shown.

Considering that *acr11* plants show abnormally high levels of amino acids and other nitrogen-containing metabolites, we probed whether this metabolic phenotype and the growth defects observed in *acr11* plants could be impacted by nitrogen availability in the environment. To this end, we grew Col-0, *acr11–1* and *acr11–2* plants on soil under medium-light conditions (i.e., ~100 µmol·m^-2^·s^-1^) watering them with 1:10 Hoagland solution for the first two weeks (1.5 mM total nitrogen provided as NO_3_^-^, see methods), then switching to four different watering solutions having 0, 30, 60 and 120 mM of total nitrogen (**Figure 5A**). After around ten days of switching to the nitrogen watering solutions, the 30 mM nitrogen treatment started causing delayed growth and necrotic leaf lesions in *acr11* knock-out lines, with the 60 mM nitrogen solution worsening this phenotype, and the 120 mM solution killing all *acr11* plants (**Figure 5A**).

Based on the preliminary experiments above, we performed the same experiment but watering the plants with 15 mM or 30 mM total nitrogen, which also caused numerous necrotic lesions on the leaves of *acr11* plants (**Supplementary S4**), although not to the severity of the 60 mM and 120 mM solutions (**Figure 5A**). After three weeks of switching to the nitrogen solutions, the whole rosette of these plants was harvested and subjected to elemental composition analysis to determine total carbon and nitrogen content. This analysis returned results not consistent between the two *acr11* mutant lines when the plants were watered with the diluted Hoagland’s 1:10 control solution, with nitrogen content increasing by a 15% in *acr11-1*, while decreasing by 15% in *acr11-2*, compared to Col-0 (**Figure 5B**). In contrast, at 15 mM nitrogen watering solution, both *acr11* lines showed a 16% increase in N content compared to Col-0, which resulted in a 15% decrease in carbon/nitrogen ratio, as carbon content remained unchanged between Col-0, *acr11–1* and *acr11-2* (**Figure 5B**). Despite previous results suggesting severe susceptibility to high nitrogen in *acr11* mutants (**Figure 5A**), Col-0, *acr11–1* and *acr11–2* plants had the same nitrogen and carbon content when watered with the 30 mM nitrogen solution (**Figure 5B**). These findings support that, in addition to experiencing severe toxicity under high nitrogen fertilization, *acr11* mutants have alterations in carbon/nitrogen balance. These alterations, however, are seemingly complex and do not necessarily correlate with the amount of nitrogen fertilization.

To test whether high carbon input may counter *acr11* disruptions, we grew Col-0, *acr11–1* and *acr12–2* plants for three weeks under medium light intensity (~100 µmol·m^-2^·s^-1^) and high-CO_2_ (~1000 ppm) to increase carbon fixation in photosynthesis. Consistent with increased photosynthesis, high-CO_2_ conditions induced both Col-0 and *acr12–2* plants to grow significantly larger than plants grown at environmental [CO_2_] (**Figure 2**), reaching ~750 milligrams of fresh weight (mg FW) total rosette mass on average (**Supplementary S5**). However, *acr11–1* plants total rosette mass was around ~65 mg FW (**Supplementary S5**), i.e. around only half the size of *acr11–1* plants grown at environmental [CO_2_] (~130 mg FW; **Figure 2A**) and less than a 10% of the average Col-0 grown side-by-side at high-CO_2_. Therefore, the reduced growth phenotype of *acr11* plants was worsened by elevated CO_2_. Taken together, these results support that knock-out mutation of *ACR11* causes severe growth and carbon/nitrogen imbalance, and that these alterations are heavily influenced by nitrogen supply.

### High nitrogen exacerbates *acr11* metabolic perturbations

**Figure 6:**
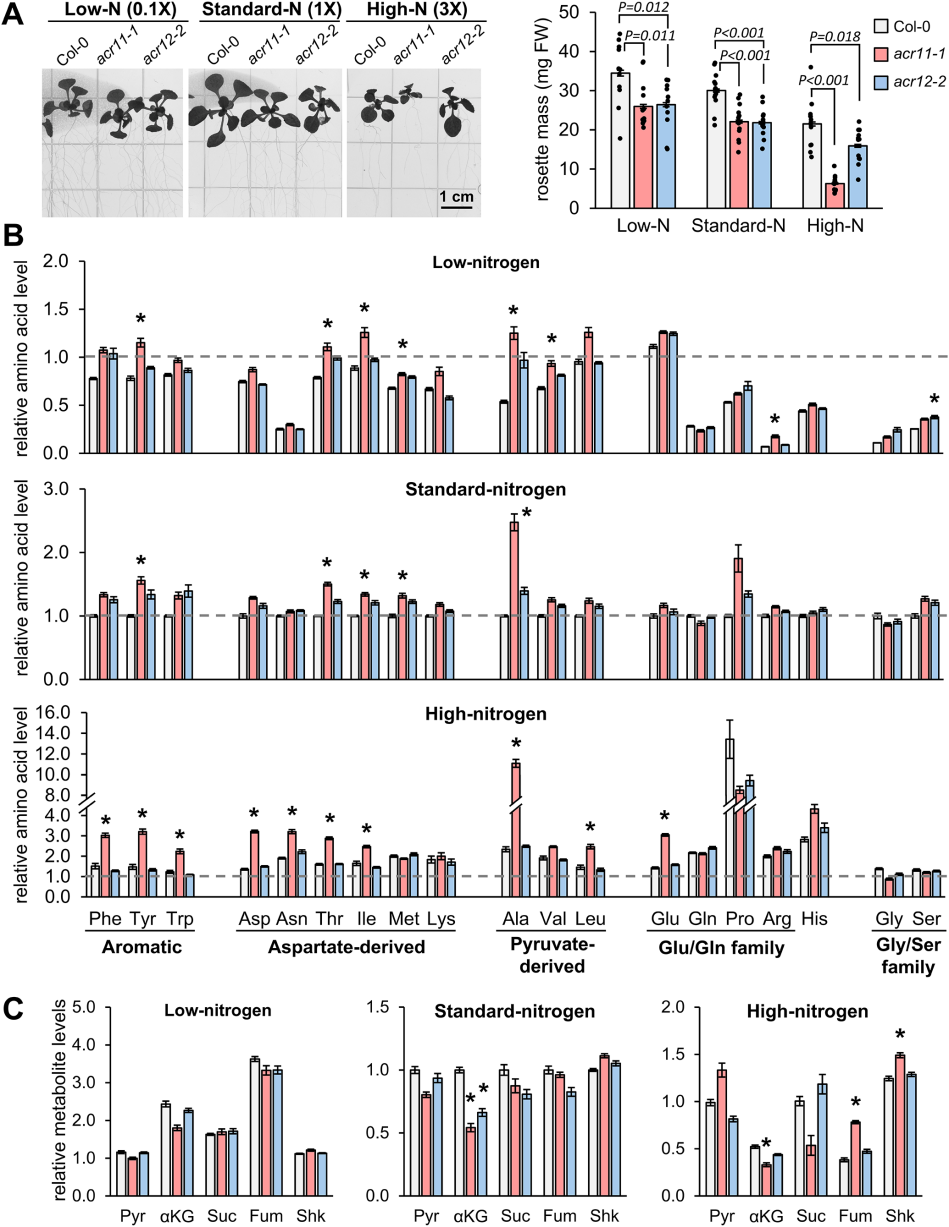
High nitrogen in the growth media exacerbates overaccumulation of free amino acids in *acr11*. **(A)** Picture and total rosette mass of two-weeks-old Col-0, *acr11–1* and *acr12–2* plants grown side-by-side in half Murashige-Skoog (½MS) media having 0.1-, 1- and 3-times the standard dose of total nitrogen in this media (see methods). LC-MS determination of **(B)** free proteinogenic amino acids and **(C)** pyruvate (Pyr), α-ketoglutarate (αKG), succinate (Suc), fumarate (Fum) and shikimate (Shk) levels in the rosettes of Arabidopsis plants grown *in vitro* under different nitrogen concentrations. Metabolite levels are expressed in relative units by assigning the value of “one” (indicated by horizontal dashed line on each graph) to the average abundance of that metabolite in Col-0 plants grown under standard-nitrogen conditions. Bar graphs represent the average of *n* = 8 samples, except *n* = 6 for pyruvate levels in *acr11–1* plants under high nitrogen (see original data in the supplemental files), each sample consisting of one individual plant. Error bars correspond to standard error. Asterisks indicate significant differences compared to Col-0 plants grown under the same nitrogen conditions, according to *post hoc* ANOVA Tukey’s test (*α* = 0.05).

To gain insight into how high nitrogen availability affects *acr11* plants metabolism, we grew *acr11–1* plants side-by-side with *acr12–2* and Col-0 on nitrogen-free ½ Murashige-Skoog (MS) media supplemented with 1% sucrose and different concentrations of nitrogen, corresponding to 0.1X, 1X and 3X the total nitrogen concentration in ½ MS media commonly used for Arabidopsis *in vitro* culture (i.e., ~3, 30 and 90 mM of total nitrogen, respectively, supplied as a mixture of NH_4_^+^ and NO_3_^-^; see Methods) for two weeks. This experiment further supported that the growth of *acr11–1* plants was severely affected by high nitrogen concentrations (**Figure 6A**). Both *acr11* and *acr12* mutants grew similarly in low- and standard-nitrogen plates, reaching around ~75% of the rosette mass of Col-0 (**Figure 6A**). In contrast, in high-nitrogen media, the rosette mass of *acr11* plants dropped to only ~30% that of Col-0, whilst *acr12* plants stayed comparatively unaffected at ~75% the size of Col-0 (**Figure 6A**). Hence, *acr11* mutants, but not *acr12*, seem to be highly sensitive to high nitrogen toxicity.

Subsequent metabolic analysis supported that, in addition to having a negative impact on *acr11–1* plant growth (**Figure 6A**), high nitrogen concentrations exacerbated the tendency of *acr11–1* plants to over accumulate free amino acids (*post hoc* ANOVA Tukey’s test, *α* = 0.05). In low-nitrogen media, six amino acids were significantly overaccumulated in *acr11* compared to Col-0: tyrosine, threonine, isoleucine, methionine, alanine, valine and arginine. Similar results were observed for *acr11* in standard-nitrogen media, except for valine and arginine (**Figure 6B**). Conversely, ten amino acids were overaccumulated in high-nitrogen media: phenylalanine, tyrosine, tryptophan (all derived from the shikimate pathway), aspartate, asparagine, threonine, isoleucine (all four belonging to the aspartate pathway), alanine, leucine and glutamate. Remarkably, fold changes between *acr11* and Col-0 were higher if compared to low- and standard-nitrogen media. This was particularly evident for alanine, which was 5-times higher in *acr11* compared to Col-0 in high-nitrogen media (**Figure 6B**), compared to ~2-fold increase in low- and standard-nitrogen media (**Figure 6B**). Further, *acr11* plants showed decreased levels of α-ketoglutarate in standard- and high-nitrogen media (**Figure 6C**), which is consistent with elevated glutamate levels in the latter (**Figure 6B**). In contrast to *acr11* plants, *acr12* mutants showed few significant differences compared to Col-0: only elevated serine in low-nitrogen media (**Figure 6B**) and reduced α-ketoglutarate in standard-nitrogen media (**Figure 6C**). These experiments revealed that overaccumulation of free amino acids in *acr11* plants is exacerbated by high nitrogen in the media.

### High nitrate or ammonium are not specifically causing a*cr11* metabolic perturbations

**Figure 7:**
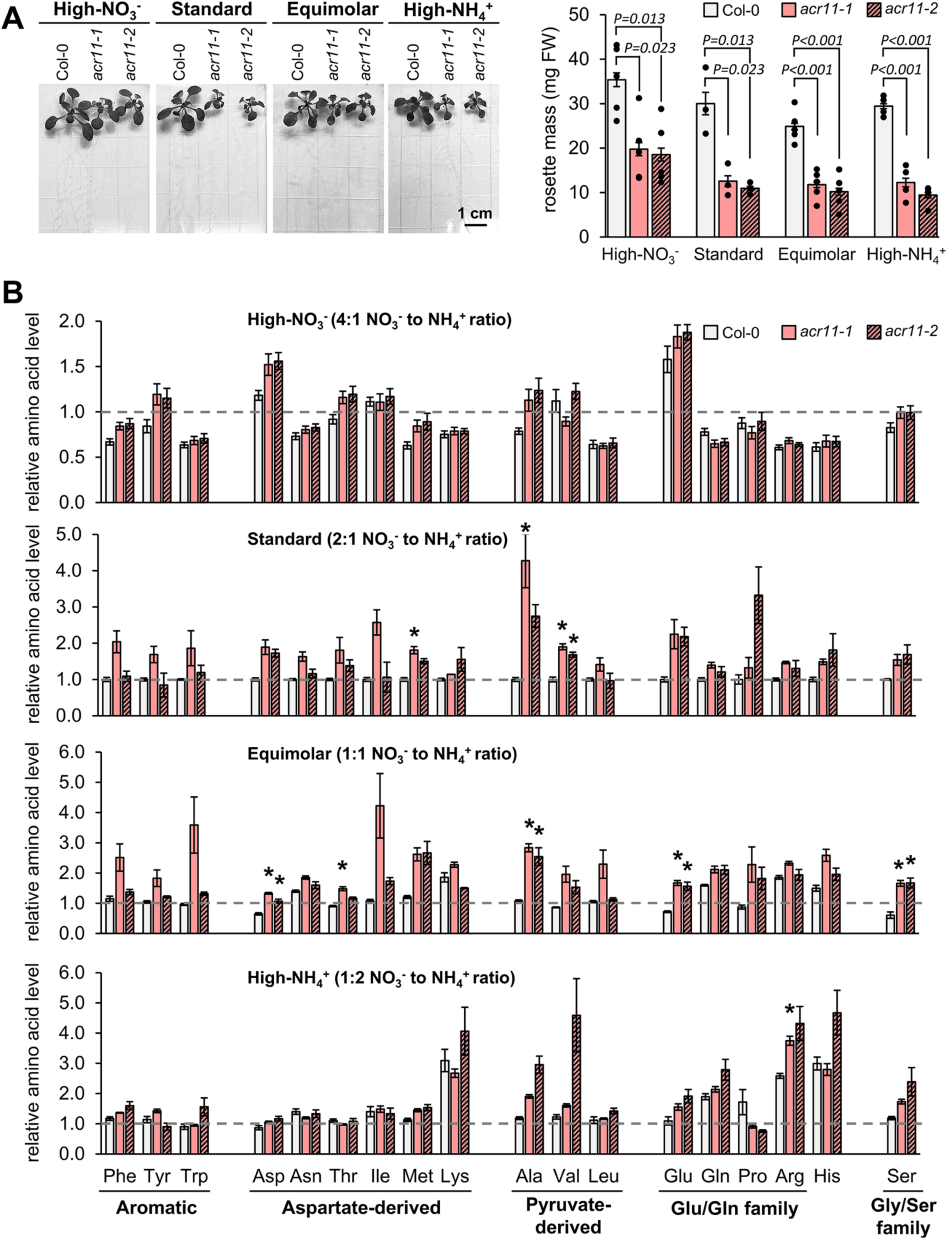
High NO_3_^-^ attenuates metabolic disruptions in *acr11*. **(A)** Total rosette mass of two-weeks-old Col-0, *acr11–1* and *acr11–2* plants grown side-by-side in half Murashige-Skoog (1/2MS) media having a constant concentration of total nitrogen (equaling regular 1/2MS media), but provided in varying NO_3_^-^ to NH_4_^+^ (NO_3_^-^/NH_4_^+^) ratios (see methods). **(B)** LC-MS determination of free proteinogenic amino acids in the different NO_3_^-^/NH_4_^+^ media used. Metabolite levels are expressed in relative units by assigning the value of “one” (indicated by horizontal dashed line on each graph) to the average abundance of that metabolite in Col-0 plants grown under standard-nitrogen conditions. Bar graphs represent the average of *n* = 2–5 samples, each sample consisting of two individual plants grown side-by-side on the same plate. Error bars correspond to standard error. Asterisks indicate significant differences compared to Col-0 plants grown under the same nitrogen conditions, according to *post hoc* ANOVA Tukey’s test (*α* = 0.05).

**Figure 8:**
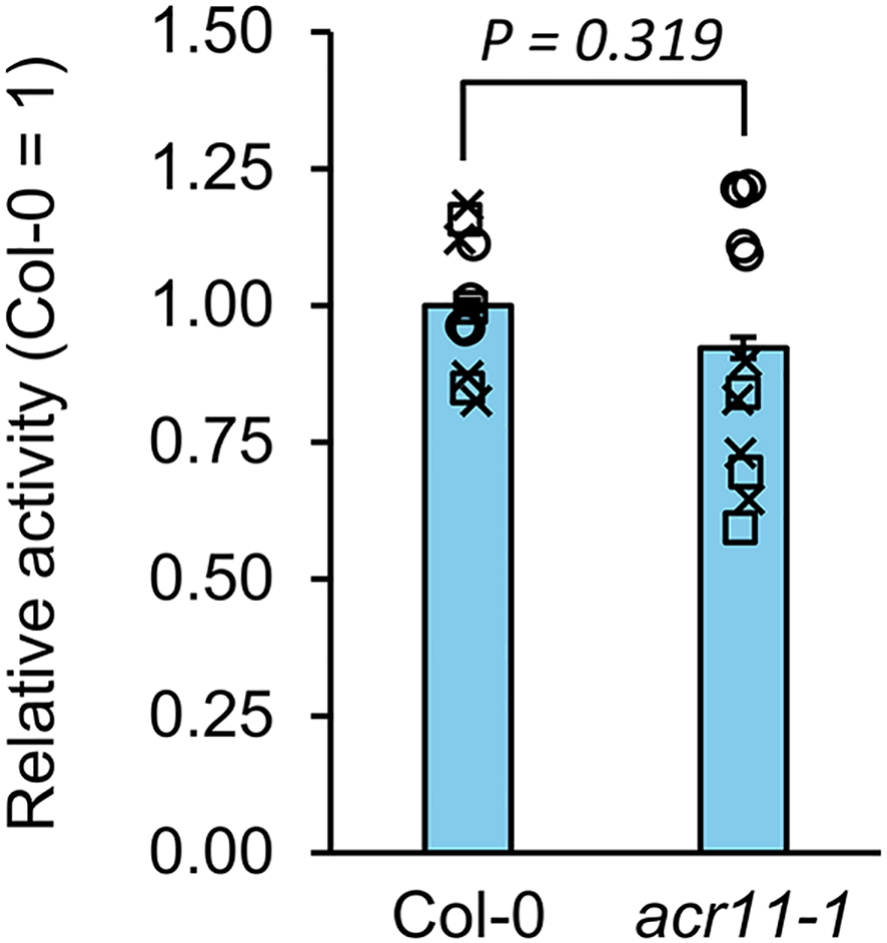
Deregulation of glutamine synthetase activity does not explain altered nitrogen metabolism in *acr11*. Glutamine synthetase (GS) activity measured in the leaves of one-moth-old Col-0 and *acr11–1* plants grown on soil under standard light conditions, and watered regularly with a diluted Hoagland solution (see methods). GS activity was measured as time-course production of ^15^N-glutamine in the presence of ATP, glutamate and ^15^NH_4_^+^ during a 30 min linear reaction, and then corrected by the mass of total proteins in the extract. Three batches of independently grown Col-0 and *acr11–1* plants were tested, which are represented as individual symbols: squares (**□**, *n* = 3), crosses (×, *n* = 4), and circles (○, *n* = 5). To compare these three independent experiments, GS activity within each set of plants was expressed in relative units based on the average GS activity level of Col-0, which was given the reference value of one. GS activity in the Col-0 plants from the three independent batches was, in nmol·min^-1^·mg protein^-1^: 7.3, for □ samples; 0.3, for × samples; and 56.7, for ○ samples. Bar graphs represent the combined average relative activity for the three independent experiments. Error bars correspond to combined standard error for the three independent experiments. *P*-value according to Student’s *t*-test based on comparing the combined relative data from the three independent experiments.

As nitrogen-rich growth media exacerbates *acr11* growth and metabolic disruptions, we sought to test if this effect could be specifically linked to excess of NH_4_^+^ or nitrate (NO_3_^-^) in the growing media. Col-0 were grown alongside two *acr11* knock-out lines, *acr11–1* and *acr11-2*, on ½ MS media containing the standard 30 mM total nitrogen for Arabidopsis *in vitro* culture, but supplied in varying NO_3_^-^:NH_4_^+^ ratios: high-NO_3_ (4:1), standard-ratio (2:1; like in regular MS media), equimolar-ratio (1:1), and high-NH_4_^+^ (1:2) (see methods). The size of *acr11–1* and *acr11–2* plants, measured as total rosette mass, indicated that *acr11* grew equally regardless of the NO_3_^-^/NH_4_^+^ ratio in the media (*P*-value = 0.08; one-way ANOVA), around 45% the rosette mass of Col-0 (**Figure 7A**). Conversely, the amino acid overaccumulation phenotype typically shown by *acr11* was significantly impacted by the NO_3_^-^/NH_4_^+^ ratio (*P*-value < 0.001; two-ways ANOVA). The *acr11* plants grown in standard- and equimolar-ratio of NO_3_^-^/NH_4_^+^ showed significant overaccumulation for three and five amino acids, respectively, in at least one of the two *acr11* lines tested (**Figure 8B**) (*post hoc* ANOVA Tukey’s test, *α* = 0.05). Although the number of significantly altered amino acids in *acr11* versus Col-0 was lower if compared to previous experiments (**Figures 3B**, **6A**) likely due to a lower number of biological replicates, the overall observations were consistent across experiments, with *acr11* plants showing a tendency to over accumulate aromatic amino acids, multiple aspartate-derived amino acids, alanine, valine, glutamate and serine (**Figure 7A**). Conversely, *acr11* plants grown on high-NO_3_^-^ media showed no significant differences with Col-0 (**Figure 7B**). On high-NH_4_^+^ media, only arginine was significantly overaccumulated in *acr11–1* compared to the Col-0, but not in *acr11-2* (**Figure 7B**). These trends suggest that overaccumulation of amino acids in *acr11* plants is enhanced when both NO_3_^-^ and NH_4_^+^ are provided at similar ratios, instead of being specifically linked to high levels of NO_3_^-^ or NH_4_^+^ only.

### *acr11* mutants with altered amino acids levels have normal levels of glutamine synthetase activity

Previous studies have proposed that ACR11 interacts with plastidial GS of plants and regulates this enzyme, similarly to the bacterial sensor protein GlnD (Osanai et al., 2017). To evaluate if overaccumulation of nitrogen metabolites in *acr11* might be due to altered GS activity, we grew Col-0 and *acr11–1* for one month on soil under medium-light and standard-nitrogen fertilization conditions (see methods), and performed GS assays in total protein extracts from leaves, where *ACR11* is highly expressed (Osanai et al., 2017). To increase the assay’s accuracy, we determined GS activity by directly measuring glutamine production by LC-MS, and desalted the plant protein extracts to remove the endogenous glutamine. Furthermore, the assays were conducted using ^15^NH_4_^+^ to confidently identify the glutamine produced by GS activity during the assay (i.e., ^15^N-glutamine).

Quantification of GS activity across three independent sets of Col-0 and *acr11–1* plants showed that, under these growth conditions, *acr11–1* mutants had no significant alteration in GS activity (**Figure 8**). Although this assay does not differentiate between cytosolic and plastidial GS isoforms, it can be assumed that most of the GS activity detected corresponds to plastidial GS, as this latter is clearly predominant over cytosolic GS in leaves and photosynthetic tissues (Tobin et al., 1985; Edwards et al., 1990; Castro-Rodriguez et al., 2011; Marino et al., 2022). Extraction and quantification of metabolites in these same plant samples showed that they still had significantly elevated levels for multiple amino acids (**Supplementary S6**), as observed in previous experiments using full rosette tissue and comparable growth conditions (**Figure 3**). These findings suggest that the altered levels of total nitrogen and nitrogen metabolites observed in *acr11* plants are not caused by altered total GS activity levels, at least under the growth conditions tested here.

## Discussion

**Figure 9:**
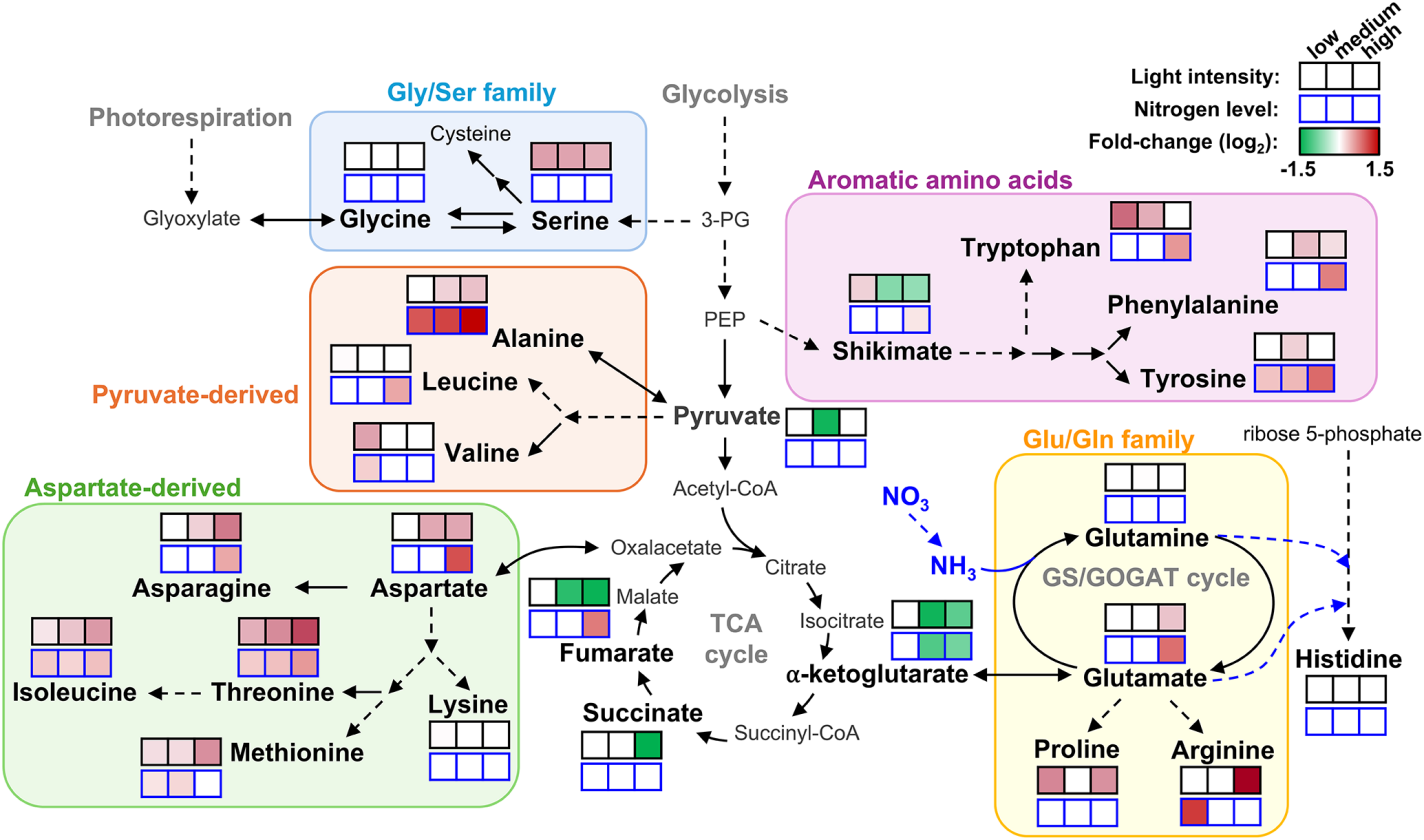
Overview of amino acid and central carbon intermediates levels in *acr11* plants under different light and nitrogen nutrition regimens. Color scale, from green to red, indicate the fold change for each measured metabolite in *acr11–1* plants compared to Col-0 under the same conditions of light intensity or nitrogen level in the media (low-, medium- and high-, from left to right), according to the metabolite results shown in main **Figures 3** and **6**, respectively. Only metabolites showed in bold were measured. No significant changes according to Tukey’s test as a white box. Discontinuous arrows indicate multiple enzymatic steps.

Metabolite profiling under different conditions of light and fertilization regimes shows that *acr11* knock-out Arabidopsis mutants overaccumulate multiple free proteinogenic amino acids in rosette tissue (**Figure 9**), where *ACR11* is highly expressed (Takabayashi et al., 2016; Sung et al., 2011; Toufighi et al., 2005). Whereas overaccumulation of free amino acids in *acr11* were previously reported (Osanai et al., 2017), our untargeted metabolomics analysis has revealed that multiple other nitrogen-rich compounds are also overaccumulated in *acr11* rosettes (**Figure 4**; **Table 1**). We also observed a sharp decrease in the TCA intermediates succinate, fumarate, and α-ketoglutarate, and the glycolytic product pyruvate, in *acr11* (**Figures 3B**, **6C**). As these metabolites are fundamental precursors to multiple amino acids (**Figure 9**), their depletion appears to be a likely consequence of excessive amino acid biosynthesis in *acr11* mutants. Conversely, loss of *ACR12* negatively impacts plant growth on MS plates (**Figure 6**) but has little effect on metabolite levels in the rosette (**Figures 3**, **6**), possibly due to low *ACR12* expression in photosynthetic tissue (Takabayashi et al., 2016; Toufighi et al., 2005). Despite the lack of any obvious significant effect on Arabidopsis *acr12* mutants, *ACR12* orthologs are conserved across all land plants (**Figure 1A**), strongly suggesting that this gene still has a relevant role in certain tissues or under certain environmental conditions.

Although *acr11* mutants consistently show reduced growth, overaccumulation of nitrogen-containing metabolites, and decreased levels of central carbon metabolism intermediates across different growth conditions, the degree of these alterations is highly influenced by the environment. For example, both low- and high-light conditions attenuated *acr11* growth defects (**Figure 2**) but had differential impact on the metabolic phenotype: low-light attenuates amino acid overaccumulation, whereas high-light exacerbates it (**Figure 3B**). Low-light conditions reduce the activity of the electron transport chain in the chloroplast and the generation of NADPH and ATP, which may constrain energy-intense processes such as nitrogen assimilation and amino acid biosynthesis (Yoneyama and Suzuki, 2020). Moreover, a recent study has shown that the expression of genes encoding enzymes involved in nitrogen assimilation decreases under low light (De Silva et al., 2025). Therefore, the attenuation of *acr11* phenotype by low light may stem from reduced nitrogen assimilation. Regarding the attenuation of *acr11* growth defects under high-light, this treatment increased amino acid levels but also the levels of central carbon metabolism intermediates (**Figure 3B**). Therefore, the high light-dependent attenuation of *acr11* growth defects may thus result from a more favorable balance between amino acids and keto acid precursors compared to medium-light conditions.

Whilst the effect of light on *acr11* growth and metabolism seems complex and possibly indirect, we observed a direct correlation between nitrogen availability in the growing media and *acr11* perturbations. Hence, high-nitrogen fertilization caused evident signs of toxicity in *acr11* plants, such as highly reduced growth, damaged leaves, and enhanced overaccumulation of free amino acids (**Figures 5**, **6**, **9**). Moreover, *acr11* mutants showed altered carbon/nitrogen balance due to higher total nitrogen content under high-nitrogen conditions (**Figure 5B**), suggesting that nitrogen metabolism is disrupted in *acr11* plants. On the other hand, we observed relatively minor metabolic alterations in *acr11* plants growth on media having either high-NO_3_^-^ or high-NH_4_^+^ (**Figure 7B**), which may be caused by reduced nitrogen uptake under these conditions (Chen et al., 2024; Straub et al., 2017; Wu et al., 2019; Hao et al., 2023). As *ACR11* expression is correlated with various photorespiratory activities (Obayashi et al., 2022), it is possible that altered carbon/nitrogen balance and free amino acid levels in *acr11* are both caused by defects in the photorespiratory pathway, which is highly interconnected with nitrogen metabolism (Florian et al., 2013; Walker et al., 2024; Shi and Bloom, 2021; Marino et al., 2022). However, *acr11* plants grown under high CO_2_ conditions, which reduce photorespiration, still showed severe growth defects (**Supplemental S5**). Collectively, these observations point out that an early step of nitrogen metabolism, such as transport or assimilation of inorganic nitrogen into amino acids, is impaired in *acr11* plants.

Prior studies postulated that ACR11 regulates plant nitrogen metabolism at the level of nitrogen assimilation in the plastidial GS/GOGAT cycle (Osanai et al., 2017; Takabayashi et al., 2016; Lee et al., 2023b). The study by Osanai and collaborators reported that ACR11 interacts with plastidial GS (encoded by *GLN2*) *in vitro* and increases the enzyme’s affinity towards its substrate, although this was determined *in vitro* using recombinant proteins and measuring GS activity indirectly through the γ-glutamyl transferase assay. Consistent with decreased GS activity in *acr11*, this study reported a ~50% reduction in free glutamine levels in *acr11–2* compared to Col-0 in plants grown for one month at ~70 µmol·m^-2^·s^-1^ light intensity under nitrogen-limiting conditions (rockwool substrate watered with 0.35 mM NO_3_^-^; Osanai et al., 2017). In an independent study, Takabayashi and collaborators found that ACR11 interacts with plastidial Fd-GOGAT *in vitro*, and that *acr11* plants have both lower levels of Fd-GOGAT protein and activity, indicating that ACR11 stabilizes Fd-GOGAT *in planta*. This second study reported markedly reduced levels of glutamate and glutamine in *acr11* plants compared to Col-0 in response to nitrogen re-supply after a nitrogen starvation period, which may be in good agreement with decreased Fd-GOGAT protein levels in this mutant (Takabayashi et al., 2016). These two studies agree on *acr11* plants having decreased levels of glutamine and/or glutamate, as well as on proposing a role for ACR11 as positive regulator of the plastidial GS/GOGAT cycle, albeit with alternative mechanisms (Osanai et al, 2017; Takabayashi et al., 2016). In our study, however, we did not observe consistently lower glutamine or glutamate content in *acr11* compared to Col-0, except for a ~25% decrease in glutamate levels under low-light which was, however, not statistically significant for this sample size (**Figure 3B**). Rather, we observed significant increases in glutamate under both high-light (**Figures 3B**, **9**) and high-nitrogen (**Figures 6B**, **9**), a quite remarkable finding given that glutamate levels in plants are usually stable across varying conditions (Forde and Lea, 2007). Importantly, metabolite levels in *acr11* were heavily influenced by environmental factors, such as light intensity, total nitrogen, and relative abundance of NO_3_^-^ and NH_4_^+^ in the media (**Figures 3**, **6**, **7**), which may explain the differences between our current findings and prior reports. Still, *acr11* plants consistently overproduce multiple amino acids and nitrogen-containing compounds (**Figures 3**, **4**, **6**), have an altered carbon/nitrogen ratio due to predominantly elevated nitrogen content (**Figure 5**), and experience severe toxicity in high nitrogen media (**Figures 5**, **6**). These findings together suggest that ACR11 might be a negative regulator of nitrogen uptake or assimilation rather than an activator.

Although our findings suggest that ACR11 may function as a negative regulator of plant nitrogen uptake or assimilation, it remains unclear whether GS/GOGAT is the target of this regulation. Total GS activity in the leaves of *acr11* plants grown on soil under standard conditions (see methods) was on the limit of statistical significance compared to Col-0 (**Figure 8**). Although our plant growth conditions are different from those used by Osanai and collaborators, who reported a ~30% decrease in GS activity in *acr11* plants grown in rockwool watered with 0.35 mM NO_3_^-^ (Osanai et al., 2017), our *acr11* plants consistently show altered levels for multiple metabolites (**Figures 3**, **4****;****Supplementary S2**, **S6**) under the standard growth conditions in which we found no altered GS activity (**Figure 8**). Thus, the observed alterations in metabolite levels in *acr11* mutants may have a different origin. The hypothesis of ACR11 as a regulator of plastidial GS activity was partly based on structural similarities between ACR11 and the bacterial regulatory protein GlnD (Osanai et al., 2017; Sung et al., 2011), which uridylates/de-uridylates the regulatory protein P_II_, which in turn regulates bacterial GS via adenylation/de-adenylation (Leigh and Dodsworth, 2007; Bolay et al., 2018). While a conserved mechanism for GS regulation between bacteria and plastids would enticingly recall the endosymbiotic origin of this organelle, this hypothesis is challenged by several lines of evidence. First, the evolutionary history of GS in the green lineage is complex, and the plastidial GS of plants has eukaryotic instead of prokaryotic origin, being thus structurally very different from bacterial GSs (Eisenberg et al., 2000; Valderrama-Martín et al., 2022). Consistent with these differences, neither adenylation nor uridylation seemingly occur in plastidial GS from plants (Leigh and Dodsworth, 2007; Xue et al., 2022). Also, our phylogenetic analysis indicates plastid-localized ACR11 is conserved across land plants (**Figure 1A**), including conifer species lacking plastidial GS (Valderrama-Martín et al., 2022; Cánovas et al., 2007), making unclear why ACR11 would retain a plastidial localization if its molecular target is no longer located within this subcellular compartment. Lastly, bacterial GlnD regulates GS only indirectly via uridylation/de-uridylation of the regulatory protein P_II_ (Huergo et al., 2013; Leigh and Dodsworth, 2007; Bolay et al., 2018). Although plants have plastidial orthologs to P_II_, plant P_II_s do not regulate GS, and instead modulate arginine biosynthesis by interacting with the enzyme with N-acetyl-L-glutamate kinase (NAGK) (Ferrario-Méry et al., 2006; Mizuno et al., 2007; Uhrig et al., 2009; Chellamuthu et al., 2014). Interestingly, Arabidopsis P_II_ mutant plants show decreased content of arginine and the arginine biosynthetic pathway intermediates L-ornithine and L-citrulline (Ferrario-Méry et al., 2006), whereas *acr11* plants show elevated levels of arginine, L-acetylornithine and L-citrulline (**Figures 3B**, **5**). Furthermore, P_II_ mutants, like *acr11* mutants, over accumulate multiple free amino acids (Ferrario-Méry et al., 2008) and are more sensitive to nitrogen toxicity (Ferrario-Méry et al., 2005). Regarding the potential stabilization of Fd-GOGAT by ACR11 *in vivo* (Takabayashi et al., 2016), reduced Fd-GOGAT activity in *acr11* plants may decrease overall nitrogen assimilation capacity and amino acid biosynthesis, leading to accumulation of toxic NH_4_^+^, which may explain the high sensitivity of *acr11* plants to excess nitrogen (**Figures 5**, **6**). However, overaccumulation of glutamate and decreased α-ketoglutarate content (**Figures 3**, **6**, **9**) are not consistent with decreased GOGAT activity—indeed, they suggest the opposite. Taken together, our current findings suggest that ACR11 may have other functions in the regulation of plant nitrogen metabolism besides controlling the GS/GOGAT cycle.

In conclusion, our findings support that ACR11 plays an important role in the regulation of plant nitrogen metabolism and carbon/nitrogen balance, possibly by influencing the relative proportion between free amino acids and other nitrogen-containing compounds versus keto acid precursors from central carbon metabolism. Although the exact molecular mechanism of ACR11-mediated regulation remains to be elucidated, our data suggest that this protein is a repressor of plastid nitrogen metabolism.

## Materials and methods

### Plant growth conditions on soil and light intensity experiments

Arabidopsis plants grown on soil under “standard conditions” were grown in individual 7×7×6.3 cm (side × side × tall) plastic pots using Jiffy’s Soilless Starting Seed Mix Number 7, under ~100 µmol·m^-2^·s^-1^ light intensity, 12h day/night cycle at 22 °C/18 °C, 70% humidity. Unless explicitly noted (see nitrogen dose experiments), plants were watered as needed with a 1:10 dilution of Hoagland’s solution in distilled water. The 1X undiluted Hoagland’s solution contained the following: 0.506 g/L KNO_3_, 1.18 g/L Ca(NO_3_)_2_·4H_2_O, 0.493 g/L MgSO_4_·7H_2_O, 0.043 g/L Fe(III)-EDTA (chelated ferric sodium salt), 0.136 g/L KH_2_PO_4_, 0.014 g/L B(OH)_3_, 0.017 g/L MnSO_4_, 0.735 mg/L CuSO_4_·5H_2_O, 1.965 mg/L ZnSO_4_·7H_2_O, and 0.55 mg/L (NH_4_)Mo_7_O_24_·7H_2_O (ammonium molybdate).

For light intensity experiments, plants were grown on soil in individual pots (7×7×6.3 cm; side × side × tall) in a growth chamber (model LED-41L2; Percival Scientific, Perry, Iowa, USA) under medium-light conditions (~100 µmol·m^-2^·s^-1^) for one week after germination. After one week, plants were divided into three groups. Plants treated with low- and high-light were then moved to ~33 and 200 µmol·m^-2^·s^-1^, respectively, whereas medium-light plants remained at 100 µmol·m^-2^·s^-1^, all within the same growth chamber. Plants were grown for three more weeks under these conditions and then harvested individually by flash-freezing in liquid nitrogen, ground to a fine powder and stored at -80°C until analysis. Environmental conditions other than light intensity were kept constant during the experiment: 12h day/night cycle at 22 °C/18 °C, 70% humidity. Plants were watered regularly with the 1:10 diluted Hoagland’s solution (see previous paragraph).

### Phylogenetic analysis of ACR11 and ACR12 sequences

ACR11 and ACR12 ortholog protein sequences, except for the gymnosperm species *Pinus taeda* (PITA), *Picea abies* (MA) and *Gnetum montanum* (Tn), were searched in Phytozome v13 using pBLAST search in the following genomes, using Arabidopsis AtACR11 or AtACR12 as bait (species abbreviations between parenthesis): *Botryococcus braunii v2.1* (Bobra) *Chlamydomonas reinhardtii v5.6* (Cre), *Chromochloris zofingiensis v5.2.3.2* (Cz), *Micromonas pusilla CCMP1545 v3.0* (Mycpu), *Ostreococcus lucimarinus v2.0* (Ostlu), *Volvox carteri v2.1* (Vocar), *Dunaliella salina v1.0* (Dusal), *Marchantia polymorpha v3.1* (Mapoly), *Physcomitrium patens v6.1* (Pp), *Sphagnum fallax v1.1* (Sphfalx), *Ceratodon purpureus GG1 v1.1* (Cepur), *Thuja plicata v3.1* (Thupl), *Selaginella moellendorffii v1.0* (Selmo), *Ceratopteris richardii v2.1* (Ceric), *Diphasiastrum complanatum v3.1* (Dicom), *Amborella trichopoda v1.0* (AmTr), *Liriodendron tulipifera YP108A v1.1* (Litul), *Nymphaea colorata v1.2* (Nycol), *Mimulus guttatus TOL v5.0* (MgTOL), *Medicago truncatula Mt4.0v1* (Medtr), *Populus trichocarpa v4.1* (Potri), *Solanum lycopersicum ITAG5.0* (Solyc), *Dioscorea alata v2.1* (Dioal), *Acorus americanus v1.1* (Acora), *Zostera marina v3.1* (Zosma), *Brachypodium distachyon v3.2* (Bradi), and *Sorghum bicolor v5.1* (Sobic). ACR11 and ACR12 orthologs from the gymnosperms *Pinus taeda* (PITA), *Picea abies* (MA) and *Gnetum montanum* (Tn) were searched by pBLAST in https://plantgenie.org/. Protein sequences without the putative plastid transit peptide were aligned using MUSCLE in MEGA-13. Phylogenies were reconstructed in MEGA-13 using Maximum-Likelihood, with site coverage cutoff set at 90%. Evolutionary distances were computed using the Poisson correction method, and are in units of number of amino acid substitutions per site. Branch support values (bootstrap) were calculated based on 200 replications.

### Identification of knockout mutant lines

The acr*11* and *acr12* mutant lines—*acr11-1* (WiscDsLoxHs054_06G), *acr11-2* (SAIL_14_H10), *acr12-1* (SAIL_594_C06), and *acr12-2* (SALKseq_05679.0)—were ordered from The Arabidopsis Information Resource (https://www.arabidopsis.org/). Naming nomenclature was followed from previous publications (Takabayashi et al., 2016). DNA for genotyping was extracted from rosette leaves following Edward’s method (Edwards et al., 1991). Knockout mutants were identified with a Polymerase Chain Reaction (PCR) using the following primers: for *ACR11*, forward primer 5’-CTTAGTTTTGTGCTGATTCGAG and reverse primer 5’-GGATCAGGAATCAAGTTCTC. For *ACR12*, forward primer 5’-GACGACGTTGTTCCAATGC and reverse primer 5’-CAGATTGATGTCGATATCGC. Transposable elements were detected with the following primers: 5’-ATTTTGCCGATTTCGGAAC (for “SALK” line), 5’-TCCTCGAGTTTCTCCATAATAATGT (for “Wisc” line), for 5’- GCCTTTTCAGAAATGGATAAATAGCCTTGCTTCC (for “SAIL” lines).

### Nitrogen dose experiments

For testing the effect of nitrogen dose on soil, plants were germinated and grown under “standard conditions” (see previous section) for two weeks in 12×12×5.5 cm (side × side × tall) plastic pots, each pot having multiple plants of the same genotype as seen in **Figure 6** and **Supplementary S3**. During this period, the plants were watered with 1:10 diluted Hoagland’s solution (see previous section). After two weeks, the plants were divided into independent trays (30 × 30 × 10 cm; side × side × tall) each tray having one pot for each genotype tested (Col-0, *acr11–1* and *acr11-2*). The individual trays were watered once per week during three additional weeks with 1 L of one of the following solutions: distilled water only (0 nitrogen treatment); 0.475 g/L KNO_3_ plus 0.412 g/L NH_4_NO_3_ (15 mM total nitrogen); 0.95 g/L KNO_3_ plus 0.825 g/L NH_4_NO_3_ (30 mM total nitrogen); 1.9 g/L KNO_3_ plus 1.65 g/L NH_4_NO_3_ (60 mM total nitrogen); 3.8 g/L KNO_3_ plus 3.3 g/L NH_4_NO_3_ (120 mM total nitrogen).

For nitrogen dose experiments *in vitro*, seeds of Col-0, *acr11–1* and *acr12–2* were germinated and grown for five days on nitrogen-free ½ MS medium (product reference 30630200-2; PlantMedia, Dublin, Ohio, USA) supplemented with 0.95 g/L KNO_3_, 0.825 g/L NH_4_NO_3_ (i.e., equivalent to the standard nitrogen dose of regular ½ MS medium), 10 g/L sucrose, 0.5 g/L 2-(N-morpholino)ethanesulfonic acid (MES) buffer pH 5.7, and 8 g/L high strength agar (product reference A20020; Research Products International, Mount Prospect, Illinois, USA) after one week, the seedlings were transferred to new plates (9 × 9 cm square plates) having around 25 mL of nitrogen-free MS media each, prepared as described in the previous sentence except for being supplemented with three alternative concentrations of KNO_3_ and NH_4_NO_3_: 0.095 g/L KNO_3_ plus 0.0825 g/L NH_4_NO_3_ (low-nitrogen, corresponding to 1:10 of the nitrogen dose of regular ½ MS medium); 0.95 g/L KNO_3_ plus 0.825 g/L NH_4_NO_3_ (standard-nitrogen, corresponding regular ½ MS medium); or 2.85 g/L KNO_3_ plus 2.475 g/L NH_4_NO_3_ (high-nitrogen, corresponding to three-times the nitrogen dose of regular ½ MS medium). Six individual plants, two for each genotype tested, were transferred to each plate and distributed evenly. After transfer, plants were grown for ten additional days and then harvested individually by flash freezing in liquid nitrogen, ground to a fine powder, and stored at -80°C until analysis. Light, temperature and photoperiod conditions were kept constant through the experiment at ~100 µmol·m^-2^·s^-1^ light intensity, with a 12h day/night cycle at 22 °C/18°C.

For [NO_3_^-^] to [NH_4_^+^] ratio experiments *in vitro*, seeds of Col-0, *acr11–1* and *acr11–*2 were sown and germinated on nitrogen-free ½ MS medium, prepared as described at the beginning of the previous paragraph, but supplemented with four different [NO_3_^-^]/[NH_4_^+^] ratios (i.e., high-NO_3_^-^, standard-, equimolar-, and high-NH_4_^+^) provided as the following salts (**Table 2**).

**Table 2:** Nitrogen formulations in the MS media for the nitrate versus ammonium ratio experiment.

	High-NO_3_^-^	Standard-ratio	Equimolar-ratio	High-NH_4_^+^
[NO_3_^-^]/[NH_4_^+^] (approx.)	4:1	2:1	1:1	1:2
NH_4_NO_3_ (g/L)	0.489	0.829	1.207	0.423
KNO_3_ (g/L)	1.805	0.950	–	0.489
KCl (g/L)	0.07	0.70	1.40	1.05
NH_4_Cl (g/L)	–	–	–	0.786
				
Total [Nitrogen] (mM)	30.0	30.0	30.0	30.0
Total [NO_3_^-^] (mM)	23.9	19.7	15.0	10.1
Total [NH_4_^+^] (mM)	6.1	10.3	15.0	19.9

Each plate (9 × 9 cm square plates), having around 25 mL of media, contained nine individual plants (three per genotype) evenly distributed. Plants were grown on the plates for two weeks under ~100 µmol·m^-2^·s^-1^ light intensity, 12h day/night cycle at 22 °C/18 °C. For harvesting, all plants of the same genotype grown in the same plate were pooled into a single sample, flash-frozen with liquid nitrogen, ground to a fine powder and stored at -80 °C until analysis.

### High CO_2_ concentration experiments

For high versus environmental [CO_2_] experiments, Col-0, *acr11–1* and *acr12–2* seeds were germinated and grown on soil in individual pots under “standard conditions” (see first subsection of methods) for one week in a growth chamber (model LED-41L2; Percival Scientific, Perry, Iowa, USA) at ~425 ppm CO_2_ (i.e., environmental CO_2_ concentration). One week after germination, [CO_2_] was increased to ~1000 ppm. Plants were kept under these conditions for three more weeks and then harvested individually. Environmental conditions other than [CO_2_] were kept constant through the experiment at ~100 µmol·m^-2^·s^-1^ light intensity (coming from white LEDs lights only), 12h day/night cycle at 22 °C/18 °C, 70% humidity.

### Extraction and targeted analysis of plant metabolites

For extraction of plant metabolites, around 20–30 mg of pulverized frozen plant tissue, or frozen ground whole rosettes in the case of two-weeks old plants grown on MS media, were resuspended into 400 µL of methanol:chloroform (2:1) extractant, containing norvaline at 100 µM as internal standard, for ~1 minute with regular vortexing, followed by centrifugation at 20,000 *g* for 5 min at room temperature. The whole supernatant was transferred to a fresh tube, mixed with 300 µL of water and 125 µL of chloroform, and spun down at 20,000 *g* for 5 min at room temperature for phase separation. The upper, aqueous phase (~500 µL) was recovered and dried using a freeze dryer overnight. The dried pellets were resuspended into 50 µL of methanol 80%, spun down at 20,000 *g* for 5 min, and the supernatant transferred to vials for injection. All reagents used for the extraction were UHPLC-MS grade, except chloroform.

Plant extracts were analyzed using Vanquish Horizon Binary UHPLC (Thermo Scientific) coupled to a Q Exactive MS (Thermo Scientific). One microliter of the sample was analyzed using an InfinityLab Poroshell 120 HILIC-Z column (2.1 × 150 mm, 2.7-μm particle size; Agilent, Santa Clara, California, USA) in a gradient of 5 mM NH_4_^+^-acetate/0.2% acetic acid buffer in water (solvent A) and 5 mM NH_4_^+^-acetate/0.2% acetic acid buffer in 95% acetonitrile (solvent B) at a flow rate of 0.45 mL/min and column temperature of 40°C. The gradient, expressed as % of phase B in A, was: 0–2 min, 94%; 2–9 min, 94–88%; 9–19 min, 88–71%; 19–20 min, 71–20%; 20–21.5 min, 20%; 21.5–22 min, 20–94%; 22–25 min, 94%. All chemicals used to prepare the mobile phases were LC-MS grade.

For targeted analysis of amino acids and other metabolites, full MS spectra were recorded between 2 and 19 min using electrospray ionization (ESI) full scan (TIC) between *m*/*z* 70–1050 switching negative/positive polarity, or in ESI positive mode only for untargeted metabolomics (see below), under the following parameters: sheath gas flow rate, 55; auxiliary gas flow rate, 20; sweep gas flow rate, 2; spray voltage, 3 kV; capillary temperature, 350 °C; S‐lens RF level, 50; resolution, 70 000; AGC target 3 × 10^6^, maximum scan time 100 msec; scan range 70-1050 *m*/*z*. Spectral data were integrated manually using Xcalibur 3.0 based on the retention time and peak area of authentic standards.

### Untargeted metabolomics analysis

One-month-old Col-0, *acr11–1* and *acr11–2* plants (n = 7 independent plants for each genotype) were grown on soil under standard conditions (see previous section in methods about growth conditions on soil). Plant tissue, corresponding to the whole plant rosette, was collected in between 7–8 hours after the beginning of the light period, frozen in liquid nitrogen and ground to a fine powder. Metabolite extraction was conducted as described for targeted analysis of plant metabolites. High-throughput integration was conducted in MZmine v4.1.0 (Schmid et al., 2023) using full-range TIC (*m*/*z* 70 to 1050) data in positive polarity collected between 2 and 19 min. For feature detection, noise threshold was set to 1.0 × 10^4^ and 2.0 × 10^3^ for MS1 and MS2, respectively. Chromatograms were built with the LC–MS chromatogram builder tool for mass features with a minimum absolute height of ≥2.0 × 10^5^ and detected in at least seven consecutive scans, with a minimum intensity of 1.0 × 10^5^ between peaks. Local minimum resolver was applied for a minimum ratio of peak top/edge of three and a maximum peak duration of 1 min. Carbon-13 isotopes were then removed using the ^13^C isotope filter tool. Features were aligned with the join aligner tool using a retention time and *m*/*z* tolerance of 0.2 min and 10 ppm, respectively. Redundant features were consolidated using the duplicate feature filter. Features present in at least two out of four mock extractions were subtracted from the feature list, except for features at least three times more abundant in the plant samples compared with the extraction mock, which were kept. Gaps in the blank-subtracted feature list were filled using the feature finder tool. After gap-filling, features not being present in at least five plant samples were removed using the feature list rows filter tool, and a correlation analysis of the remaining features was performed in metaCorrelate. The final features list was then exported as both molecular networking files and SIRIUS outputs with merged MS2 spectra. The exported feature list was further analyzed in MS excel, the integrated peak area divided by the mass of the plant sample (in mg FW), and the recovery factor of isovitexin (determined in a manual integration) to perform statistical analysis across the dataset. The SIRIUS output file was used to predict feature identity and structure in SIRIUS v5.8.6 (Dührkop et al., 2019) for [M + H], plus [M + K] and [M + Na] as fallback adducts. ZODIAC (Ludwig et al., 2020) was enabled at default parameters to improve the search. A structure search was performed with the CSI: FingerID (Dührkop et al., 2015) in all available databases.

### Carbon and nitrogen elemental analysis

Determination of total elemental carbon and nitrogen in the plant samples was determined by combustion elemental analysis using an Elementar Vario MACRO cube CHNS analyzer, as described by Yan et al., 2022. Approximately 2.5 mg of dried and finely ground plant tissue, corresponding to the whole rosette tissue, were used for the analysis.

### Measurement of glutamine synthetase activity

Plants for glutamine synthetase (GS) activity assay were grown on soil for one month under “standard conditions” (see section above about plant growth conditions). Fully expanded leaves were harvested using liquid nitrogen and ground to a fine powder. For protein extraction, ~20 mg FW of ground frozen plant tissue was resuspended in 100 µL of ice-cold extraction buffer containing: 50 mM sodium phosphate pH 7.5, 100 mM KCl, 20 mM MgCl_2_, 2 mM ethylene diamine tetra acetic acid (EDTA), 1 mM dithiothreitol (DTT), 10% (v/v) glycerol, 0.1% (v/v) triton X-100, 1 mM PMSF (added right before use) and 1% (v/v) Protease Inhibitor Cocktail VI (Dot Scientific Inc.; added right before use). Resuspended plant tissue was kept on ice for ~2–3 min with regular vortexing, and then spun down at 15,000 g for 10 min at 4 °C. After centrifugation, the supernantant (~100 µL) was desalted twice using Zeba™ Spin Desalting Columns 7K MWCO 0.5 mL (Thermo Scientific) previously equilibrated with extraction buffer without PMSF. The desalted protein extracts were aliquoted into fractions, frozen immediately into liquid nitrogen, and stored at -80°C until the GS assay was conducted (typically in the next ~48 hours after protein extraction).

GS assay conditions were based on Rhodes et al., 1975. Reactions were carried out in a total reaction volume of 50 µL having: 35 mM imidazole buffer pH 7.2, 21 mM MgCl_2_, 15 mM ATP, 20 mM of ^15^N-labeled NH_4_Cl_2_, and 10 uL of the desalted protein fractions (which corresponds to approximately 10 to 20 µg of total proteins per reaction). Reactions mixtures with the plant extracts were prepared on ice and then incubated at 30°C for 2 min before starting the reaction by adding glutamate to a final concentration of 30 mM. Reactions were kept at 30°C and stopped by adding 50 µL of pure methanol after 10, 20, 30 and 40 min to confirm linear kinetics, followed by spinning down at 13,000 g for 2 min. Mock reactions with plant extract but no glutamate substrate, and glutamate substrate without plant extract, were run in parallel as negative control to account for unspecific or contaminating sources of glutamine. GS activity was determined by LC-MS by measuring production of ^15^N-glutamine using an InfinityLab Poroshell 120 HILIC-Z PEEK-coated column (2.1 × 50 mm, 2.7-μm particle size; Agilent, Santa Clara, California, USA) and the mobile phases A and B described above for the analysis of plant metabolites at a flow rate of 0.75 mL/min. The gradient, expressed as % of phase B in A, was: 0–0.25 min, 100%; 0.25–2 min, 100–85%; 2–3 min, 85–70%; 3–4 min, 70–50%; 4–4.2 min, 50–20%; 4.2–4.8 min, 20%; 4.8–5.0 min, 20–100%; 5.0–6.0 min, 100%. Quantification of ^15^N-glutamine was done by manual integration in Xcalibur 3.0 using a calibration curve generated with pure authentic unlabeled glutamine.

## Data Availability

The original contributions presented in the study are included in the article/**Supplementary Material**, further inquiries can be directed to the corresponding author/s.
