# Supplementary material for: Loss of Arabidopsis ACR11 results in altered C/N balance and high sensitivity to nitrogen toxicity
Source: Front Plant Sci. 2025 Dec 1;16:1679652. doi: 10.3389/fpls.2025.1679652 (PMC12702975; doi:10.3389/fpls.2025.1679652)
Supplement: Supplementary file 1 [file Supplementaryfile1.pdf]

Supplemental material for

Loss of Arabidopsis ACR11 Results in Altered C/N Balance and High Sensitivity to Nitrogen Toxicity

Kunkel JR, Maeda HA, and El-Azaz J

2025

The following supplemental materials and figures are included in this document:

Supplemental S1. Predicted subcellular localization prediction in ACR11 and ACR12 ortholog proteins across the plant phylogeny.

Supplemental S2. Free proteinogenic amino acids content in the rosette tissue of one-month-old Col-0, *acr11-1* and *acr11-2* plants grown under standard conditions.

Supplemental S3. Validation of L-citrulline and L-acetylornithine content using authentic standards.

Supplemental S4. Plants used for carbon/nitrogen analysis after 3 weeks watering with nitrogen solutions.

Supplemental S5. Total rosette mass in Col-0, *acr11* and *acr12* plants grown at 1000 ppm CO_2.

Supplemental S6. Analysis of free proteinogenic amino acid levels in Col-0 and *acr11-1* tissue samples used to measure GS activity.

	Sequence ID	Pred.	OTHER	SP	mTP	cTP	lTP	Clavage Position
**AtACR12 orthologs**	AtACR12_AT5G04740.1	cTP	0.00	0.00	0.00	0.95	0.04	CS pos: 68-69. RVY-AS. Pr: 0.3093
	Medtr1g112170.1	cTP	0.00	0.00	0.00	1.00	0.00	CS pos: 49-50. VYA-SS. Pr: 0.4629
	Potri.010G238300.1	cTP	0.18	0.00	0.00	0.80	0.01	CS pos: 66-67. ILY-AS. Pr: 0.4021
	Potri.010G248700.2	cTP	0.01	0.00	0.00	0.99	0.00	CS pos: 53-54. IPG-AS. Pr: 0.2813
	MgTOL.F0933.1	cTP	0.00	0.00	0.01	0.99	0.00	CS pos: 52-53. VCA-SV. Pr: 0.4744
	Bradi4g42320.1	cTP	0.00	0.00	0.00	0.99	0.00	CS pos: 45-46. VCQ-SI. Pr: 0.4230
	Sobic.008G037800.1	cTP	0.00	0.00	0.00	1.00	0.00	CS pos: 46-47. ICC-QS. Pr: 0.2660
	Zosma04g24250.1	cTP	0.02	0.00	0.01	0.96	0.01	CS pos: 71-72. PTA-SS. Pr: 0.2196
	Litul.01G108600.1	OTHER	0.91	0.00	0.00	0.00	0.08	
	AmTr_v1.0_scaffold00114.8	cTP	0.17	0.00	0.29	0.54	0.00	CS pos: 59-60. CRC-SS. Pr: 0.4289
	Nycol.K00179.1	cTP	0.03	0.00	0.32	0.64	0.00	CS pos: 60-61. VSR-TS. Pr: 0.2465
	Thupl.29381896s0003.1	OTHER	0.98	0.00	0.02	0.00	0.00	
	Solyc11T000191.1	cTP	0.02	0.00	0.06	0.90	0.02	CS pos: 61-62. VSC-TL. Pr: 0.7357
	Acora.05G014200.1	mTP	0.05	0.00	0.57	0.38	0.00	CS pos: 64-65. DML-GS. Pr: 0.0950
	Acora.11G036900.1	mTP	0.01	0.00	0.91	0.08	0.00	CS pos: 68-69. DFF-GS. Pr: 0.2521
	Acora.11G036600.1	mTP	0.20	0.00	0.62	0.18	0.00	CS pos: 30-31. RCF-PC. Pr: 0.1169
	Dioal.05G006600.1	cTP	0.34	0.02	0.22	0.42	0.00	CS pos: 82-83. SIN-ST. Pr: 0.2798
**AtACR11 orthologs**	AtACR11_AT1G16880.1	cTP	0.05	0.00	0.00	0.95	0.00	CS pos: 59-60. KIR-AS. Pr: 0.3277
	AmTr_v1.0_scaffold00039.223	OTHER	1.00	0.00	0.00	0.00	0.00	
	PITA_09594	cTP	0.01	0.00	0.00	0.98	0.00	CS pos: 57-58. VNN-SA. Pr: 0.3752
	Thupl.29382174s0020.1	cTP	0.04	0.00	0.35	0.60	0.01	CS pos: 55-56. SCF-SG. Pr: 0.3856
	TnS000824839t20	cTP	0.01	0.00	0.02	0.97	0.00	CS pos: 63-64. LYC-SG. Pr: 0.2818
	AmTr_v1.0_scaffold00019.297	cTP	0.00	0.00	0.00	1.00	0.00	CS pos: 51-52. RIQ-AA. Pr: 0.3534
	Nycol.D00095.1	OTHER	0.77	0.00	0.07	0.16	0.00	
	Potri.008G010400.1	cTP	0.00	0.00	0.01	0.98	0.00	CS pos: 53-54. IVQ-AS. Pr: 0.5207
	Potri.008G020700.1	cTP	0.25	0.00	0.03	0.71	0.01	CS pos: 74-75. DLN-AS. Pr: 0.0618
	Zosma05g26730.1	cTP	0.01	0.00	0.01	0.98	0.00	CS pos: 56-57. VCA-SA. Pr: 0.4165
	Medtr4g007140.1	cTP	0.03	0.00	0.17	0.79	0.00	CS pos: 66-67. PRA-AA. Pr: 0.5048
	Medtr2g105090.1	cTP	0.00	0.00	0.00	0.95	0.05	CS pos: 65-66. IPR-AT. Pr: 0.4554
	Solyc03T003067.1	cTP	0.02	0.00	0.03	0.95	0.00	CS pos: 66-67. STA-AV. Pr: 0.1836
	Solyc06T001791.1	cTP	0.00	0.00	0.00	1.00	0.00	CS pos: 57-58. VLK-AS. Pr: 0.2931
	MgTOL.I0711.1	cTP	0.00	0.00	0.00	0.97	0.02	CS pos: 65-66. ASS-AT. Pr: 0.2015
	Litul.14G019700.1	cTP	0.01	0.00	0.00	0.99	0.00	CS pos: 58-59. SPA-AV. Pr: 0.3736
	Litul.12G022000.1	cTP	0.28	0.00	0.01	0.70	0.00	CS pos: 61-62. VPN-TV. Pr: 0.1998
	Acora.01G019300.1	cTP	0.00	0.00	0.04	0.94	0.02	CS pos: 46-47. SSA-AL. Pr: 0.3489
	Dioal.16G049900.1	cTP	0.01	0.00	0.01	0.98	0.00	CS pos: 51-52. SVA-VN. Pr: 0.2158
	Dioal.09G007500.1	cTP	0.02	0.00	0.02	0.95	0.01	CS pos: 61-62. PKA-AS. Pr: 0.3074
	Bradi3g18820.1	cTP	0.00	0.00	0.00	1.00	0.00	CS pos: 55-56. PRA-AS. Pr: 0.5060
	Sobic.001G252000.1	cTP	0.00	0.00	0.00	0.99	0.00	CS pos: 56-57. VPR-AT. Pr: 0.2756
**Conifer sequences, *incertae sedis***	PITA_36487	OTHER	1.00	0.00	0.00	0.00	0.00	
	PITA_49123	cTP	0.03	0.00	0.02	0.95	0.00	CS pos: 52-53. SCM-AL. Pr: 0.4556
	MA_121279g0010	cTP	0.01	0.00	0.01	0.98	0.00	CS pos: 56-57. SCM-AL. Pr: 0.4482
	TnS000146153t03	cTP	0.10	0.00	0.19	0.71	0.00	CS pos: 61-62. LCQ-VS. Pr: 0.3398
**Bryophytes and fern sequences, *incertae sedis***	Mapoly0046s0069.1	cTP	0.22	0.00	0.26	0.50	0.03	CS pos: 82-83. AVA-SV. Pr: 0.1683
	Pp6c9_4550V6.1	cTP	0.01	0.00	0.00	0.99	0.00	CS pos: 75-76. VCC-AS. Pr: 0.5167
	Pp6c15_5440V6.1	cTP	0.01	0.00	0.00	0.99	0.00	CS pos: 93-94. VCR-AT. Pr: 0.4231
	Sphfalx12G068100.1	cTP	0.00	0.00	0.00	1.00	0.00	CS pos: 67-68. VCY-AS. Pr: 0.7184
	CepurGG1.7G072600.1	cTP	0.00	0.00	0.01	0.99	0.00	CS pos: 67-68. VCR-AS. Pr: 0.5927
	Selmo101399	OTHER	1.00	0.00	0.00	0.00	0.00	
	Selmo97374	OTHER	1.00	0.00	0.00	0.00	0.00	
	Ceric.1Z306900.1	OTHER	0.98	0.00	0.02	0.00	0.00	
	Ceric.39G039300.1	OTHER	0.63	0.01	0.36	0.01	0.00	
	Ceric.01G055000.1	cTP	0.00	0.00	0.00	1.00	0.00	CS pos: 62-63. FQH-AV. Pr: 0.4129
	Ceric.1Z004500.1	cTP	0.00	0.00	0.00	1.00	0.00	CS pos: 67-68. ACC-AM. Pr: 0.5266
	Ceric.15G045200.1	cTP	0.18	0.00	0.24	0.58	0.00	CS pos: 59-60. VVQ-EA. Pr: 0.4029
	Dicom.09G020000.1	cTP	0.02	0.00	0.11	0.85	0.02	CS pos: 54-55. DCR-AS. Pr: 0.3972
	Dicom.02G047200.1	OTHER	0.63	0.00	0.37	0.00	0.00	
	Dicom.06G098500.1	OTHER	0.36	0.00	0.31	0.33	0.00	
**Algal ACR11/12 orthologs**	Mycpu174238	cTP	0.00	0.00	0.17	0.83	0.00	CS pos: 34-35. LRA-TA. Pr: 0.2912
	Ostlu6465	OTHER	1.00	0.00	0.00	0.00	0.00	
	Cre01.g061077.t1.1	cTP	0.00	0.00	0.00	1.00	0.00	CS pos: 57-58. VCR-AA. Pr: 0.5743
	Vocar.0001s0455.1	cTP	0.00	0.00	0.03	0.97	0.00	CS pos: 59-60. VCR-AS. Pr: 0.7226
	Dusal.0701s00007.1	cTP	0.01	0.00	0.01	0.96	0.02	CS pos: 72-73. RVC-AS. Pr: 0.4399
	Cz09g15120.t1	cTP	0.23	0.00	0.10	0.67	0.00	CS pos: 63-64. LRA-SS. Pr: 0.1869
	Bobra.0108s0027.1	cTP	0.01	0.00	0.00	0.98	0.00	CS pos: 79-80. LRA-AA. Pr: 0.3502

Supplemental S1 (previous page). Predicted subcellular localization prediction in ACR11 and ACR12 ortholog proteins across the plant phylogeny. cTP, chloroplast transit peptide; mTP, mitochondria transit peptide.

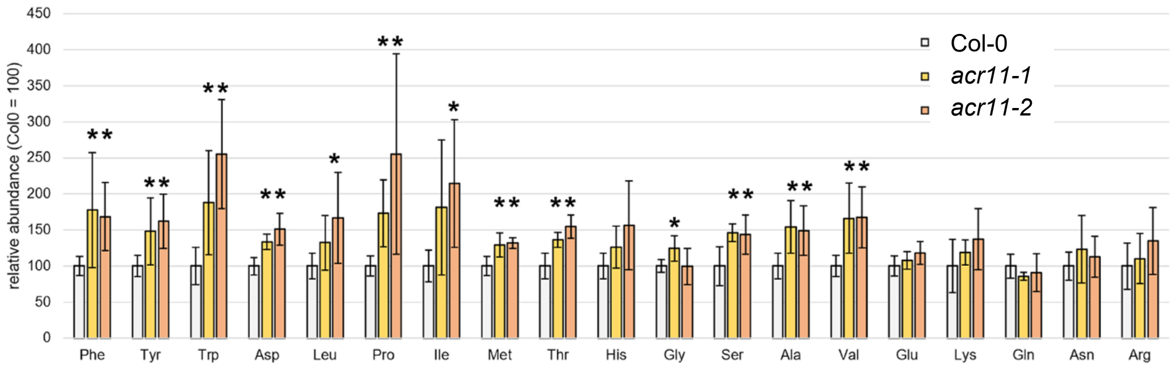

Supplemental S2. Free proteinogenic amino acids content in the rosette tissue of one-month-old Col-0, *acr11-1* and *acr11-2* plants grown under standard conditions. Plants were grown for one month on soil under standard conditions (~100 µmol·m^{-2}·s^{-1} light intensity, 12/12h photoperiod, regular watering with 1:10 Hoagland's solution; see composition in methods). Amino acid levels are expressed in relative units, with 100 corresponding to the average content of that amino acid in Col-0 plants.

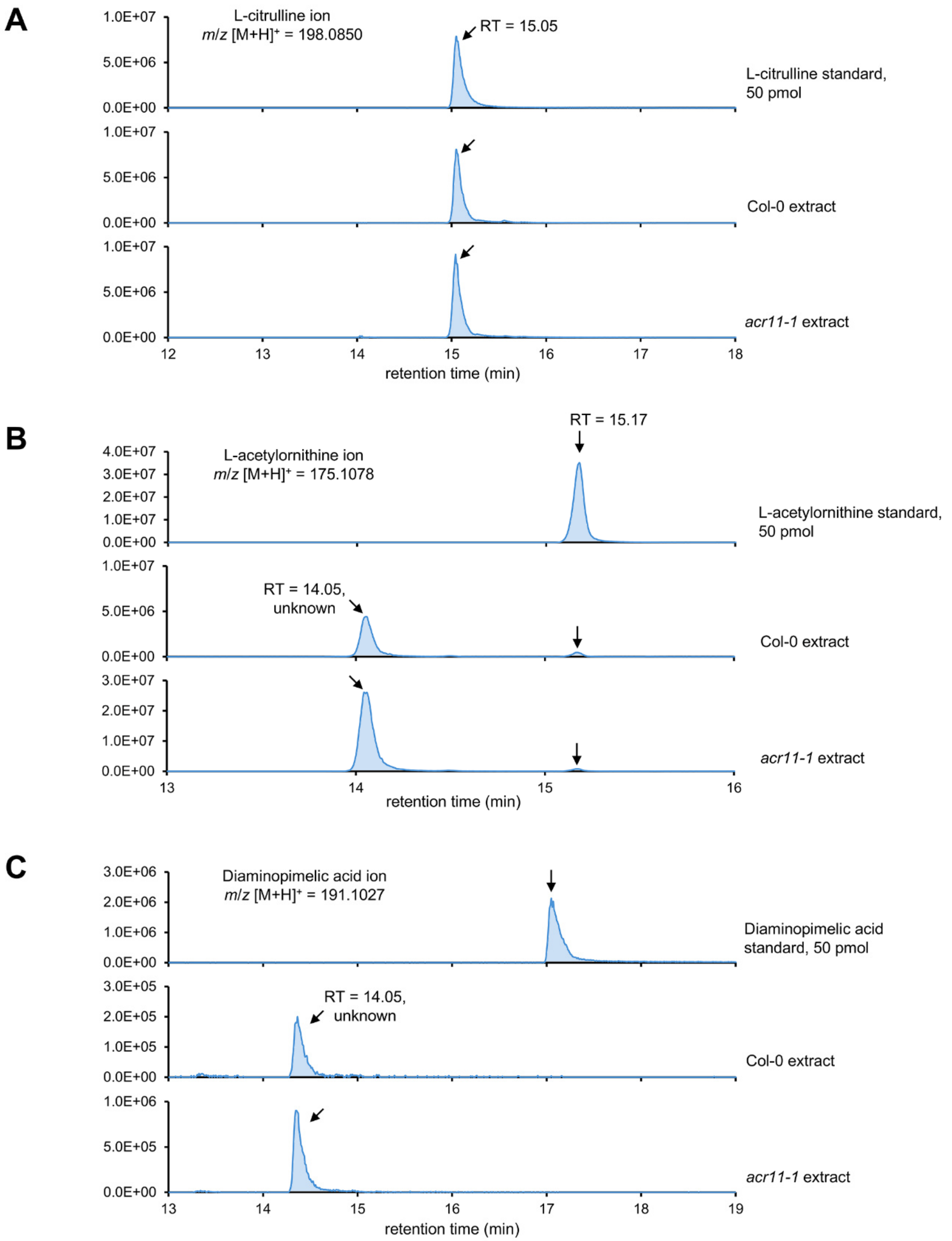

Supplemental S3. Validation of L-citrulline and L-acetylornithine content using authentic standards. Chromatograms correspond to the extracted ion for **A)** L-citrulline, **B)** L-acetylornithine and **C)** diaminopimelic acid. Note that the validated L-acetylornithine ion was found at retention time (RT) 15.17 in the plant samples. The "L-acetylornithine like" compound found at 14.05 remained unidentified.

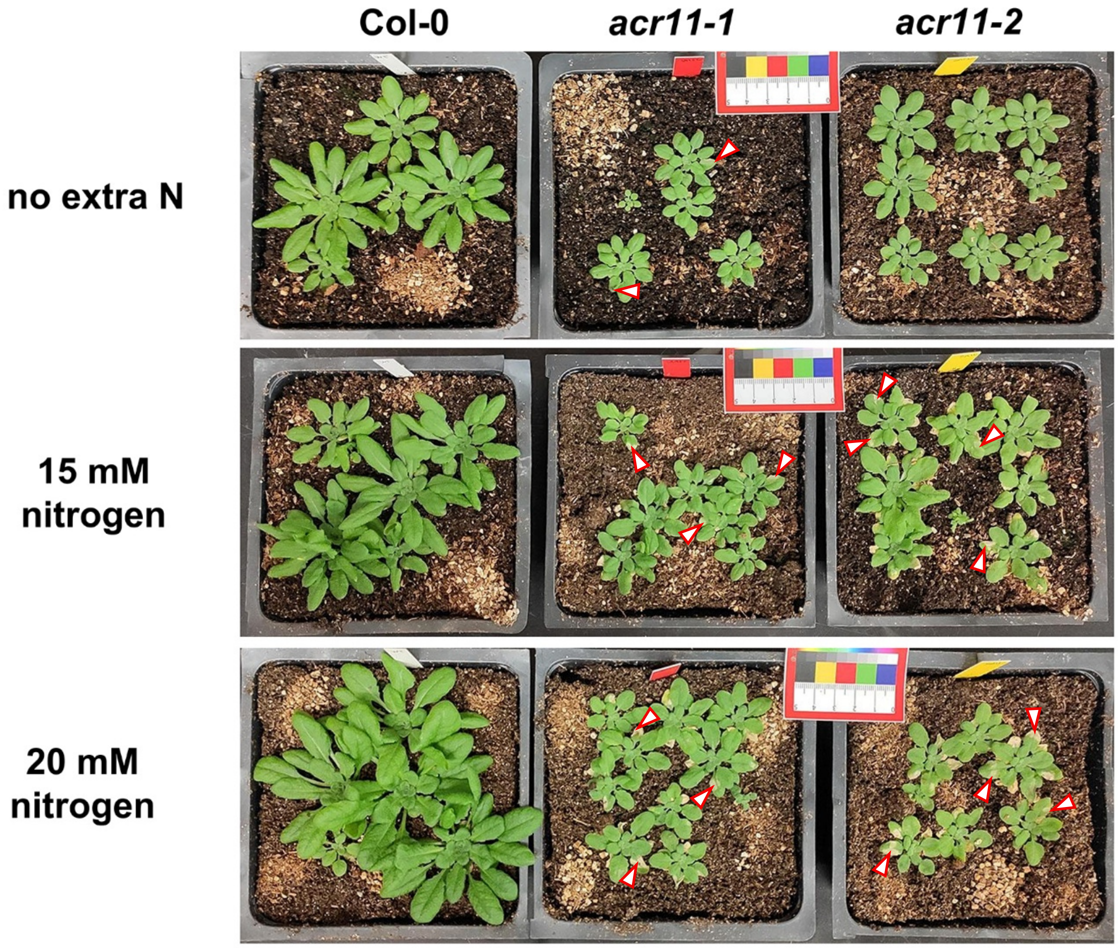

Supplemental S4. Plants used for carbon/nitrogen analysis after three weeks watering with nitrogen solutions. Plants were grown for five weeks on soil under ~100 $\mu mol \cdot m^{-2} \cdot s^{-1}$ light intensity, 12/12h photoperiod, watered regularly with 1:10 Hoagland's solution (see composition in methods) for the first two weeks. Starting on the third week, watering solution was switched to the same diluted Hoagland's solution but supplemented with additional nitrogen provided as a mixture of KNO_3 and NH_4NO_3 (see methods) for another three weeks (until a total age of five weeks). Red arrows point to leaf damage often observed in *acr11* plants, particularly at high nitrogen doses.

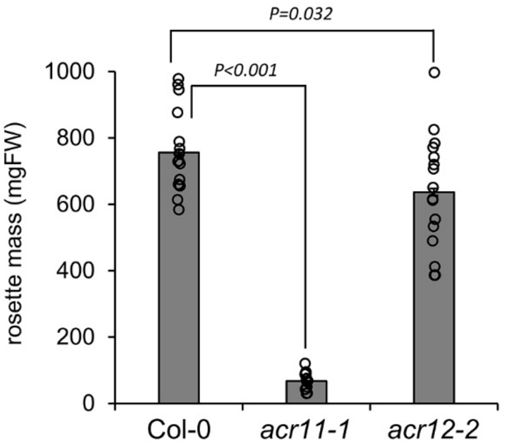

Supplemental S5. Total rosette mass in one-month-old Col-0, *acr11* and *acr12* plants grown at 1000 ppm CO_2 under ~100 µmol·m^{-2}·s^{-1} light intensity, 12/12h photoperiod, watered regularly with 1:10 Hoagland's solution (see composition in methods). Bars correspond to the average of n=16 plants. Error bars correspond to standard error. *P* values according to Student's *t*-test (two-tails test).

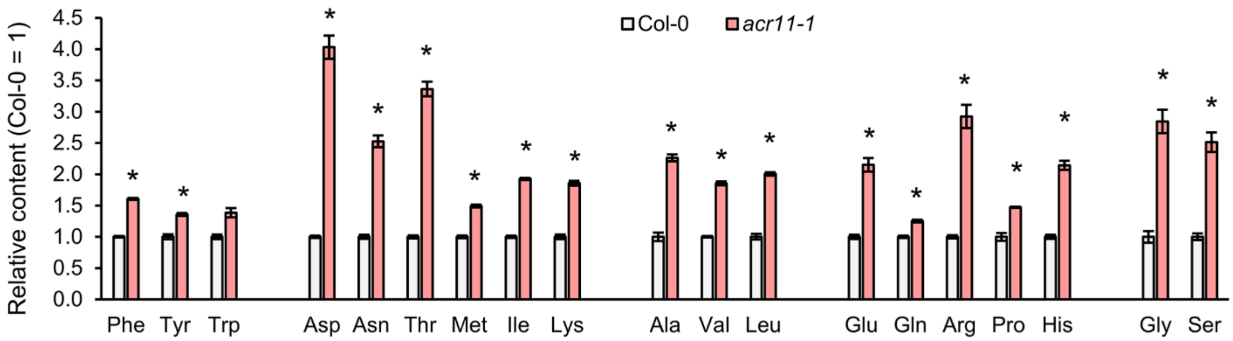

Supplemental S6. Analysis of free proteinogenic amino acid levels in Col-0 and *acr11-1* tissue samples used to measure GS activity (main Figure 8, individual samples marked with circles). The samples correspond to ground frozen expanded leaves from one-month-old plants grown on soil under standard conditions (i.e. light intensity ~100 $\mu mol \cdot m^{-2} \cdot s^{-1}$, 12/12h photoperiod, watered regularly with diluted Hogland's solution, see details in methods). Bars correspond to the average of *n*=5 individuals for Col-0 and *n*=4 for *acr11-1* due to insufficient amount of tissue left in one of the *acr11-1* plants after extracting proteins for the GS assay. Data expressed in relative units, with Col-0 corresponding to "one". Error bars correspond to standard error. Asterisks indicate statistically significant differences between *acr11-1* and Col-0 according to Student's *t*-test (two-tails test).
